# The impact of flooding on firm performance and economic growth

**DOI:** 10.1371/journal.pone.0271309

**Published:** 2022-07-13

**Authors:** Xiaofei Pan, Buhui Qiu

**Affiliations:** 1 Discipline of Finance, Faculty of Business and Law, University of Wollongong, Wollongong, NSW, Australia; 2 Discipline of Finance, University of Sydney Business School, The University of Sydney, Sydney, NSW, Australia; National Institute of Public Finance and Policy, INDIA

## Abstract

Using comprehensive flood data from China, we find a significant, negative impact of flooding on firm performance, which is mainly driven by unexpected flooding. We use multiple identification strategies to address endogeneity concerns and find that the documented impact of flooding on firm performance is likely causal. The impact is more pronounced for firms with more tangible asset investment, firms located in cities with low government quality, firms facing tight financial constraints, firms controlled by non-government entities and firms with low geographic diversification. Flood-exposed firms react to the threat by altering their investment, financial, cash, payout and executive compensation policies. Finally, flooding also exerts a significant impact on local economic and employment growth.

## Introduction

This paper employs comprehensive flood data from China to investigate the impact of flooding on firm performance, firm policies and economic growth. Due to the significant climate changes such as global warming in the past decades, environmental risk—flooding in particular—has increasingly become a threat to sustainable economic growth and human lives [1]. In particular, our colleagues [1] estimate that with an increase of 1.5°C in temperature, global human losses from flooding could rise by 70–83% and direct flood damage by 160–240%. However, the extant literature provides little empirical evidence on how flooding impacts firm performance, alters firm policies, and affects economic growth. As a country with a vast geographic area, the largest population in the world, a relatively fast-growing economy and large floods occurring almost every year, China provides us an ideal laboratory to study the actual impact of flooding on firm performance, firm policies, as well as economic and employment growth. For example, China represents approximately 18% of global people currently exposed to flooding and accounts for approximately 12% of global flood mortality [1].

We construct a comprehensive flood dataset from China covering the 2003–2019 period. We estimate panel regression models relating various firm performance measures in year *t+*1 to flood ratio of the firm’s location city in year *t*. We define flood ratio as the area of flooding divided by the total area of the city in a year. We control for a battery of firm and managerial characteristics, city-level economic development, and firm and year fixed effects. We find that flood ratio is significantly and negatively related to future firm performance in year *t*+1 including Tobin’s Q and return on assets (ROA), indicating that flooding negatively impacts firm performance. Moreover, to alleviate the concern for the ‘endogenous control’ problem [2, 3], we show that this finding is insensitive to whether we include the control variables in the regressions or not. In terms of economic magnitudes, a one-standard-deviation increase in the flood ratio is on average related to a 1.14% decrease in Tobin’s Q and a 2.36% decrease in ROA, relative to the respective sample medians of these firm performance measures. Results are qualitatively similar when we use future stock returns and return on sales as performance measures.

As flood ratio is arguably exogenous to the firm, controlling for firm fixed effects, year fixed effects, firm and managerial characteristics and city-level economic development, should be sufficient to identify the impact of flooding on future firm performance. Nevertheless, given that poor public choices, inefficient crisis management, corruption and incompetence of a city’s management system and/or other omitted factors may lead to both significant flooding and poor firm performance, we adopt multiple identification strategies to address such endogeneity concerns.

First, in order to investigate whether local economic conditions and crisis management abilities affect our baseline results, in each year we match each treatment (flood-exposed) firm with a control (non-flood-exposed) firm that operates in the same industry as the treatment firm and is located very close to the treatment firm to form a matched sample. The rationale behind this empirical strategy is that firms located very close to each other should face very similar local economic conditions and local crisis management abilities. We then reestimate the baseline regression results using this matched sample. The regression results are very similar to the baseline results, again suggesting that the impact of flooding on firm performance is most likely causal and not driven by unobserved local economic conditions or crisis management abilities.

Second, since some floods are likely to be more predictable than others, we examine whether sudden floods and expected floods have differential impacts on future firm performance. We categorize flooding of a city-year into sudden flooding if the flood ratio of the city in the year is two-standard-deviation larger than the average flood ratio of that city during our sample period, and categorize flooding into normal flooding otherwise. Sudden flooding is less likely to be expected and prepared for by residents. Thus, it should have stronger impact on future firm performance than normal flooding. Indeed, we find that the negative impact of flooding on future firm performance is mainly driven by sudden flooding and not by normal flooding. Third, we check how sensitive the OLS estimates are to omitted variables using the approach of our colleagues [4] and find that the results are robust. We further employ the instrumental-variable regression approach developed by our colleague [5]. The results are also robust to this instrumental-variable regression approach.

Our subsample regression results reveal that the negative impact of flooding on firm performance is more pronounced for firms with more intensive tangible asset investment, likely because tangible capital assets such as plants, equipment and warehouses can suffer significant damages due to flooding. Moreover, the negative effect of flooding on firm performance is also stronger for firms located in cities with low government quality, indicating that poor public choices and inefficient/incompetent city management can exacerbate the negative impact of flooding.

Because financially constrained firms do not have enough financial resources to absorb the negative shocks occasioned by flooding, such firms may suffer more in crisis times. For example, during the Global Financial Crisis, financially constrained firms planned deeper cuts in technology spending, employment, and capital spending, burned through more cash, drew more heavily on lines of credit, sold more assets to fund their operations, and bypassed attractive investment opportunities [6]. Consistent with this notion, we find that the negative impact of flooding on firm performance is significantly greater for firms facing tight financial constraints. Furthermore, state-owned enterprises (SOEs) are more likely to receive various government financial supports and subsidies than non-state-owned enterprises (non-SOEs) [7, 8]. Thus, SOEs should be better able to cushion the negative flood shocks. Indeed, we find that the negative impact of flooding on firm performance mainly concentrates in non-SOEs.

In addition, we partition the sample firms based on their levels of geographic dispersion. We find that future performance of firms with greater geographic dispersion (as proxied by the number of cities the firm’s subsidiaries are located in) is less affected by flooding, suggesting that geographic diversification is important to mitigate the negative impact of flooding. We further document that the impact of flooding on firm performance is more severe in firms without insurance compensation than in firms with insurance compensation. Finally, we find that the flood effect on long-term future firm performance is statistically insignificant, indicating that the impact of flooding on firm performance is relatively short-run in nature.

Next, we turn to firms’ policy reactions to the negative impact of flooding. In terms of investment policy, we find that flood-exposed firms increase their intangible research and development expenditures (R&D) but reduce their tangible capital expenditures (CAPEX). Although intangible knowledge assets and tangible capital assets can both improve firm productivity, the latter is far more vulnerable to flood threat than the former. Our finding that flood-exposed firms increase R&D but decrease CAPEX is thus consistent with such firms substituting intangible assets for tangible assets in their production processes to mitigate the impact of flooding.

Interestingly, we further find that firms threatened by flooding are more likely to announce mergers and acquisitions (M&A) and spend more on acquiring other firms. Importantly, such firms are more likely to acquire target firms located in cities unaffected by flooding rather than target firms located in flood-affected cities. This finding indicates that flood-exposed firms try to acquire targets located in non-flooded areas and shift their operations to the target firms to mitigate the impact of flooding. This evidence is also consistent with our prior subsample results based on geographic dispersion which suggest that the negative impact of flooding can be mitigated by geographic diversification of firm production.

Short-term debt can lower firm value if it has to be refinanced at high interest rate due to capital market frictions [9–12]. Moreover, firms tend to hold cash reserves for precautionary reason [13]. Looking at financial and cash policies, we find that flood-exposed firms tend to increase their financial leverage and lengthen debt maturity. They also tend to increase their cash reserves to cushion the negative flood impact.

Cash dividends are “sticky” and represent a commitment to shareholders [14]. Firms with permanent operating cash flows tend to pay dividends, while firms with temporary cash flows tend to repurchase shares [15–17]. Looking at payout policy, we find that flood-exposed firms have lower dividend payout ratio and are less likely to pay cash dividends. By contrast, they have higher share repurchase ratio and are more likely to repurchase shares. Overall, our empirical evidence indicates that firms threatened by flooding try to increase their financial flexibility by altering their financial, cash and payout policies to mitigate the impact of flooding.

In terms of executive compensation policy, we find that flood-exposed firms decrease their CEO pay, indicating that firm managers also bear personal risk of flooding. Moreover, flood-affected firms decrease their CEO pay-performance sensitivity, which is reasonable given that flood-exposed firms’ inferior performance relative to their non-affected industry peers is at least partly attributable to flooding that is outside of managerial control.

Finally, we examine the impact of flooding on local economic and employment growth, as the impact of flooding on local firm performance may aggregate into an impact on local economic and employment growth. Using city-level panel regressions with control for local economic conditions as well as city and year fixed effects, we document that flooding is significantly and negatively related to future employment growth and economic growth of the affected city. In terms of economic magnitudes, a one-standard-deviation increase in flood ratio is on average related to a 1.38-percentage-point decrease in employment growth and a 0.08-percentage-point decrease in GDP growth of the city. The evidence suggests that flooding has posed a significant threat to local economic development in China.

This study is related to the scant literature studying the impact of environmental risks on firms. Our colleagues [18] find that firms in European regions impacted by flooding on average show higher total assets and employment growth than firms in regions unaffected by flooding and such positive flood effects are more pronounced for firms with higher shares of intangible assets. Combining daily data on temperatures across the continental U.S. with detailed establishment data, our colleagues [19] find no evidence that temperature exposures affect establishment-level or firm-level sales or productivity. Using a global index that captures a country’s aggregate loss due to extreme weather events, our colleagues [20] find that firms in countries suffering greater loss due to extreme weather have lower profitability and more volatile earnings and hold more cash reserves.

We contribute to this literature by documenting and quantifying the impact of flooding on firm performance and firm policies based on new, comprehensive empirical evidence from China, an under-investigated country with a vast geographic area, the second largest economy and the largest population in the world. We find that flooding exerts significantly negative and likely causal impact on future firm performance and firms react to the threat by altering their investment, financial, cash, payout, and compensation policies to mitigate the negative impact of flooding.

Our study is also related to the cross-country literature that examines the impact of climate changes on a country’s economic growth and performance [21–24]. This literature shows that climate changes can significantly impact a country’s economic performance and growth. We study the impacts of flooding on firm performance and economic and employment growth within a single country—China. Because China has a vast geographic area but the same historical origin, political regime, a dominance of the Han population, and a high Mandarin Chinese penetration rate, we can avoid potential confounding effects of culture, language and political system, and focus on the impact of flooding. We contribute to the literature by documenting new empirical evidence indicating a significant, negative impact of flooding on local employment and economic growth.

The remainder of the paper is organized as follows. Section 2 describes the data and sample and provides the summary statistics of the variables used in the study. Section 3 presents the empirical results on the impact of flooding on firm performance. Section 4 presents the empirical results on firms’ policy reactions to the threat of flooding. Section 5 reports the results on the impact of flooding on local employment and economic growth. Section 6 provides concluding remarks. The Appendix provides the definitions of all variables used in the study and their data sources, as well as additional empirical results.

## Data and sample

### Data description and sample construction

We start our sample construction with all the public firms listed on Shenzhen Stock Exchange and Shanghai Stock Exchange from 2003 to 2019. Firm financial data and managerial compensation data are obtained from the Chinese Stock Market and Accounting Research (CSMAR) database. Our initial sample consists of 35,373 firm-year observations between 2003 and 2019. Then, following the conventional data-cleaning procedure, we exclude 782 firm-year observations from financial industry, 1,939 firm-year observations flagged with either ST or ST* (note that ST stands for Special Treatment, which indicates that a firm has had negative net income for the past two consecutive years; ST* indicates that a firm has had negative net income for the past three consecutive years), 123 firm-year observations with negative book equity value, and 2,115 firm-year observations with missing information. Our final sample contains 30,414 firm-year observations corresponding to 2,891 unique firms. The headquarters of these firms spread across 247 unique cities in China (note that for Chinese listed firms, the registration location is always the same as the headquarter location).

To study the effects of flooding on firm performance and firm policies, we next combine the firm-year panel data with comprehensive flood panel data of these 247 cities which we manually collect from several sources according to the following procedure. First, we manually search the Urban Statistical Yearbooks of all these 247 cities (including the 4 province-level municipalities) to obtain the data on the area (measured in square metres) affected by flooding in each city and each year. Then, we complement and cross-validate the flood data by searching the Provincial Statistical Yearbooks of all provinces (including the 5 autonomous regions) in China, which report the area (measured in square metres) affected by flooding in each city-year in that particular province. Both the Urban Statistical Yearbooks and Provincial Statistical Yearbooks are official publications recording various statistics compiled by various levels of Chinese governments.

If there is missing information from the Urban Statistical Yearbooks and Provincial Statistical Yearbooks, we try to obtain the missing data by searching the website of the National Disaster Reduction Centre (http://www.ndrcc.org.cn/), which provides the information on natural disasters in China, including the flood area at the city-year level. Finally, the Natural Disaster dataset of the CSMAR database reports the agriculture area affected by flooding at the city-year level and the total flood area at the province-year level (including the 4 province-level municipalities and the 5 autonomous regions). If the flood area of a city-year is still missing after the above-described procedure, we fill in the missing data with the agriculture area affected by flooding from CSMAR (note that we fill in the missing data with the agriculture area affected by flooding for 45 city-year observations, which only account for about 1.5% of the city-year observations in the sample). We further cross-validate our flood area data at the city-year level with the flood area data at the province-year level to ensure the data accuracy and consistency. We further collect the information on the total area of each city and its unique city code using Baidu search engine, which allows us to calculate the proportion of each city area affected by flooding in each year during our sample period.

CSMAR provides the data on headquarter addresses of public firms and the location cities of public firms’ headquarters. We merge the firm-year data from CSMAR with our hand-collected city-year flood data using headquarter city name and unique city code.

### Summary statistics

**Fig 1:**
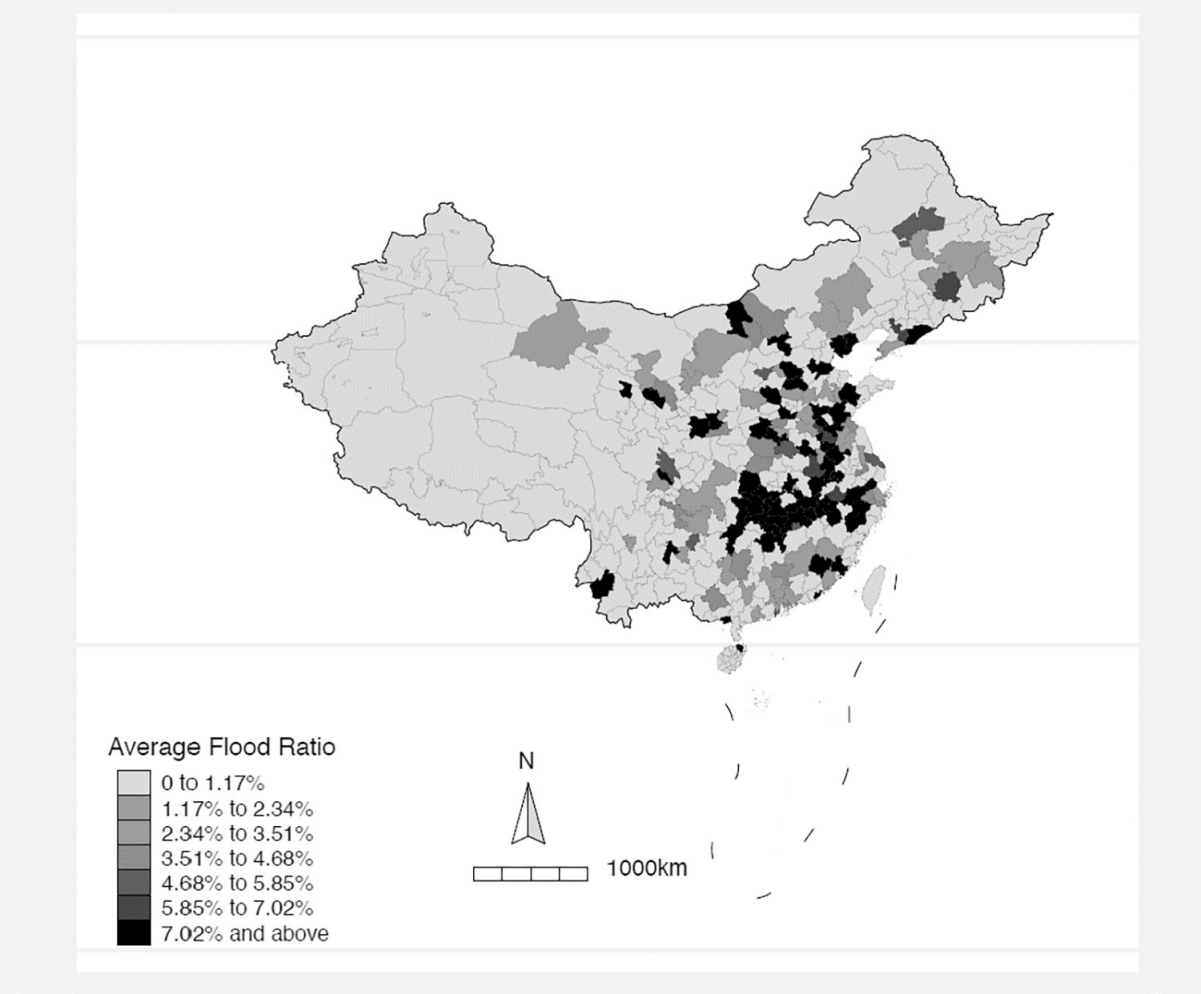
Flood ratio across mainland China. The figure plots the average flood ratio of each city across mainland China over the sample period of 2003–2019. The average flood ratio is calculated as the ratio of a city’s flood area to its total area. All Chinese cities are equally divided into seven groups according to the average flood ratio of a city and displayed in the map, with more flood-exposed cities shown in darker color. Average flood ratio is reflected in percentage in the map.

Fig 1 visualizes how flooding affects different cities across China. The darker the color in the figure, the higher the average flood ratio (i.e., the flood area divided by the total area) of the city over the 2003–2019 period. The figure clearly shows that flooding is a serious threat in the eastern part of China, in which most Chinese economic activities concentrate. The issue of flooding is especially severe in the Yangtze river region, one of the most important economic regions in China.

Table 1 shows the summary statistics of all variables used in this study. All continuous variables are winsorized at both top and bottom 1% levels. In particular, the table shows that the average performance measures of the sample firms is 2.46 for Tobin’s Q (*Tobin’s Q*), 12.9% for annual stock returns (*Stock returns*), 3.2% for return on assets (*ROA*), and 6.2% for return on sales (*ROS*). In terms of firm investment policies, our sample firms on average spend 2.2% of total assets on intangible research and development expenditures (*R&D*), 7.7% on tangible capital expenditures (*CAPEX)*, and 2.3% on mergers and acquisitions expenditures (*MA_Exp*). On average, 29.1% of the sample firms conduct M&A in any given year (*MA*) and 16.8% (12.3%) choose target firms from non-flood areas (from flood areas) (*MA_nonflood* and *MA_flood*).

**Table 1:** Summary statistics. This table shows the summary statistics of all the variables used in this study. The sample period is from 2003 to 2019. Firm-year observations from financial industry, flagged with either ST or ST*, with negative equity value are excluded. The final sample has a total of 30,414 firm-year observations and 2,891 unique firms. Variable definitions are in Table A1 in the S1 Appendix.

	N	Mean	Median	Std. Dev.	10%	90%
***Flood area***						
Floodratio	30,414	0.002	0	0.015	0	0.008
Floodratio_sudden	30,414	0.002	0	0.014	0	0.008
Floodratio_normal	30,414	0.001	0	0.001	0	0.001
***Firm performance***						
Tobin’s Q	29,808	2.459	1.878	1.799	1.092	4.465
Stock returns	30,414	0.129	0.001	0.662	-0.343	0.755
ROA	29,635	0.032	0.033	0.066	-0.007	0.095
ROS	29,635	0.062	0.060	0.199	-0.013	0.219
***Firm investment policies***						
R&D	16,317	0.022	0.018	0.018	0.002	0.043
CAPEX	27,384	0.077	0.042	0.843	0.005	0.155
MA	25,093	0.291	0	0.455	0	1
MA_nonflood	25,093	0.168	0	0.374	0	1
MA_flood	25,093	0.123	0	0.328	0	1
MA_exp	25,093	0.023	0	0.036	0	0.081
***Firm financing policies***						
Book leverage	28,808	0.449	0.449	0.209	0.163	0.721
Market leverage	28,808	0.276	0.228	0.205	0.048	0.586
Debt maturity	28,808	0.098	0.020	0.150	0	0.314
Cash holding	28,772	0.163	0.126	0.129	0.041	0.341
***Firm payout policies***						
Dividend	28,808	0.686	1	0.464	0	1
DPS	27,164	0.109	0.050	0.238	0	0.140
DPS/Book per share	26,753	0.025	0.013	0.088	0	0.059
Repurchase ratio	28,808	0.040	0	0.356	0	0
Repurchase	28,808	0.084	0	0.281	0	0
***Managerial compensation***						
CEO pay	27,383	14.101	14.123	0.810	13.081	15.059
***Other variables***						
Market-to-book	30,414	1.882	1.716	2.797	0.511	3.260
Size	30,414	21.955	21.779	1.307	20.483	23.666
Leverage	30,414	0.443	0.444	0.209	0.158	0.717
Largest	30,414	0.361	0.341	0.155	0.176	0.578
Ln(Board)	30,414	2.274	2.303	0.186	2.079	2.485
Indep	30,414	0.368	0.333	0.055	0.333	0.429
SOE	30,414	0.459	0	0.498	0	1
OCF	30,414	0.041	0.041	0.068	0.009	0.111
Tangibility	30,414	0.235	0.199	0.173	0.035	0.490
Sales growth	28,822	0.216	0.129	0.511	-0.168	0.647
CEO age	30,414	48.653	49	6.449	40	57
CEO tenure	30,414	2.918	2.342	2.638	0.422	6.599
Volatility	30,414	0.033	0.028	0.040	0.019	0.048
Subsidiary	28,389	15.122	9.000	21.866	2	32
***City level variables***						
Floodratio	3,299	0.003	0	0.016	0	0.007
Employment growth	3,299	0.125	0.032	0.417	-0.099	0.339
GDP growth	3,299	0.124	0.113	0.081	0.055	0.224
GDP	3,299	9.727	9.825	1.007	8.356	11.005
Income per capita	3,299	9.881	9.993	0.901	8.718	11.013

In relation to financing policies, the average book leverage (average market leverage) of the sample firms is 44.9% (27.6%) (*Book leverage* and *Market leverage*). On average, long-term debt accounts for 9.8% of total debt (*Debt maturity*), and cash holding accounts for 16.3% of total assets (*Cash holding*). With respect to payout policies, 68.6% of the sample firms make dividend payment (*Dividend*), and the average dividend per share is 0.109 (*DPS*). On average 8.4% of the sample firms repurchase their common shares (*Repurchase*) and share repurchase accounts for an average of 4.0% of equity market value (*Repurchase ratio*).

In terms of the control variables, the average market-to-book ratio of the sample firms is 1.88 (*Market-to-book*). The largest shareholder on average holds 36.1% of share ownership (*Largest*). On average 45.9% of the sample firms are controlled by various levels of Chinese governments (*SOE*). The average CEO age is 48.7 years (*CEO age*) and average CEO tenure is 2.92 years (*CEO tenure*). Our sample firms on average have operations in 15 cities across China (*Subsidiary*).

## The effects of flooding on firm performance

### Baseline empirical results

We begin our analysis by studying the impact of flooding on firm performance using the following Ordinary Least Squares (OLS) regression specification:

$${Performance}_{ict+1}=\alpha_{1}{Floodratio}_{ct}+\alpha_{2}{Size}_{ict}+\alpha_{3}{Leverage}_{ict}{{+\alpha }_{4}{Largest}_{ict}+\alpha_{5}Ln(board)}_{ict}+{\alpha_{6}{Indep}_{ict}+\alpha_{7}SOE}_{ict}{{+\alpha }_{8}{OCF}_{ict}+\alpha_{9}Tangibility}_{ict}+{\alpha_{10}{Cash\;holding}_{ict}+\alpha_{11}CEO\;age}_{ict}{{+\alpha }_{12}{CEO\;tenure}_{ict}+\alpha_{13}Volatility}_{ict}+{\alpha_{14}{Market-to-book}_{ict}+\alpha_{15}Past\;stock\;returns}_{ict}+{\alpha_{16}GDP}_{ct}+\alpha_{17}{Income\;per\;capita}_{ct}+{\alpha_{18}GDP\;growth}_{ct}+{Firm}_{i}+{Year}_{t}+\varepsilon_{it}$$ (1)

In Eq (1), *i* indicates the firm, *c* indicates the city in which firm *i* is headquartered, and *t* indicates the year. *Performance* is a measure of firm performance. Following the corporate finance literature [25, 26], we focus on two commonly used corporate performance measures: Tobin’s Q (*Tobin’s Q*) and return on assets (*ROA*). We use *Tobin’s Q* to capture market valuation of the firm. *Tobin’s Q* is calculated as the ratio of firm market value to book value of total assets. Firm market value is the sum of book value of short-term and long-term debt and market value of common shares. We use *ROA* to capture the firm’s financial performance. *ROA* is defined as the ratio of net income to total assets.

Our main independent variable of interest, *Floodratio*, is the ratio of the flood area of the firm’s headquarter city to the city’s total area. The higher the *Floodratio*, the more likely that the firm’s operations are affected by flooding. We do not use the absolute flood area of the firm’s headquarter city due to the large variations in city areas. For example, in our sample, the smallest city is Yima with 112 square metres, while the largest city is Alxa League with 270,000 square metres.

We also include a set of control variables in Eq (1) following previous studies [27, 28]. Specifically, *Size* is defined as the natural logarithm of firm total assets. *Leverage* is defined as the ratio of total debt to total assets. *Largest* is defined as the proportion of share ownership held by the firm’s largest shareholder. *Ln(board)* is the natural logarithm of total number of directors on board. *Indep* is the proportion of independent directors on board. *SOE* is an indicator variable which equals 1 if a firm is ultimately controlled by the government and equals 0 otherwise. During our sample period, 190 sample firms have changed their ultimate owners from private entities to the government, and 269 firms have changed their ultimate owners from the government to private entities. Therefore, we include both the SOE dummy and firm fixed effects in the regressions. *OCF* is the operating cash flow, defined as the ratio of net income plus depreciation to total assets. *Tangibility* is defined as the ratio of tangible assets to total assets. *Cash holding* is the ratio of the amount of cash holding to total assets. *CEO age* is the natural logarithm of CEO age, and *CEO tenure* is the natural logarithm of the number of years as the firm’s CEO. *Volatility* is the standard deviation of daily stock returns. *Market-to-book* is the ratio of market value of equity to book value of equity.

We include three time-varying, city-level control variables to capture local economic development which may affect firm performance: *GDP* (i.e., the natural logarithm of GDP, measured at the city-year level), *Income per capita* (i.e., the natural logarithm of annual income per capita, measured at the city-year level), and *GDP growth* (i.e., the change of GDP from previous year / GDP of previous year, measured at the city-year level). These variables reflect the local economic conditions such as the level of local economic development (*Income per capita*), market size (*GDP*), and growth prospect (*GDP growth*).

We also include firm fixed effects to control for any time-invariant firm heterogeneity and year fixed effects to control for any nationwide shocks that could affect firm performance during our sample period. As it takes some time for the impact of flooding to be reflected in firm performance, we measure firm performance in year *t*+1, and measure *Floodratio* and firm-level control variables in year *t*. That is, the independent variables are effectively lagged one year to match with the dependent variable in the regression model. We cluster standard errors at the city-by-year (i.e., city-year) level to account for the possibility that flooding in a city may affect multiple firms at the same time, leading to potential within-city-year correlation of the regression residuals. The findings remain qualitatively unchanged when we cluster standard errors at the firm level, however.

Table 2 presents the estimation results of Eq (1). Columns 1 and 3 report the results from the regressions without including the control variables (but with firm and year fixed effects). Columns 2 and 4 report the regression results with the control variables. We find that the coefficients of *Floodratio* are negative and statistically significant across all the regressions using *Tobin’s Q* and *ROA* as dependent variables, indicating that flooding has a significant, negative effect on firms’ one-year-ahead future market valuation and financial performance. Specifically, the OLS regression results suggest that firms affected by flooding have lower future market valuation and poorer future financial performance. In terms of economic magnitudes, column 1 (column 3) suggests that a one-standard-deviation increase in the flood ratio is on average related to a 1.14% decrease in one-year-ahead *Tobin’s Q* (a 2.36% decrease in one-year-ahead *ROA*), relative to the median *Tobin’s Q* (*ROA)* in the sample.

**Table 2:** Flooding and firm performance. This table reports the results of the effects of flooding on firm performance using various proxies for firm performance, including *Tobin’s Q* and *ROA*, which are measured in year *t+*1. Columns (1) and (3) report the regression results without the control variables and columns (2) and (4) report the regression results with control variables. The key independent variable is *Floodratio*, which is defined as the ratio of flood area in each city (where the firm’s headquarter is located) to the city’s total area and measured in year *t*. All of the control variables used in this table are also measured in year *t*. Variable definitions are in Table A1 in the S1 Appendix. Robust standard errors are clustered at the city-year level and reported in parentheses. *, ** and *** indicate the significance levels at 10%, 5% and 1%, respectively.

Dependent	(1)	(2)	(3)	(4)
Variables	Tobin’s Q		ROA	
Floodratio	-1.429**	-0.779**	-0.052*	-0.055**
	(0.570)	(0.382)	(0.031)	(0.027)
Size		-0.842***		-0.013***
		(0.056)		(0.001)
Leverage		0.579***		0.014***
		(0.088)		(0.005)
Largest		0.136		0.052***
		(0.091)		(0.006)
Ln(board)		0.128**		-0.002
		(0.064)		(0.004)
Indep		0.186		0.007
		(0.178)		(0.010)
SOE		-0.119***		-0.004
		(0.042)		(0.003)
OCF		3.202***		0.281***
		(0.307)		(0.013)
Tangibility		-0.246***		0.014***
		(0.081)		(0.005)
Cash holding		0.525***		0.064***
		(0.110)		(0.005)
CEO age		0.007		0.001
		(0.069)		(0.004)
CEO tenure		-0.055***		0.000
		(0.012)		(0.001)
Volatility		2.449***		0.014**
		(0.405)		(0.006)
Market-to-book		0.125*		0.000
		(0.070)		(0.001)
Past stock returns		0.219***		0.003***
		(0.028)		(0.001)
GDP		-0.167		-0.006
		(0.333)		(0.013)
Income per capita		0.442		0.011
		(0.281)		(0.009)
Past GDP growth		-0.236		-0.004
		(0.239)		(0.008)
Observations	29,808	29,808	29,635	29,635
Adjusted R-squared	0.624	0.728	0.384	0.443
Year fixed effects	Yes	Yes	Yes	Yes
Firm fixed effects	Yes	Yes	Yes	Yes

**Fig 2:**
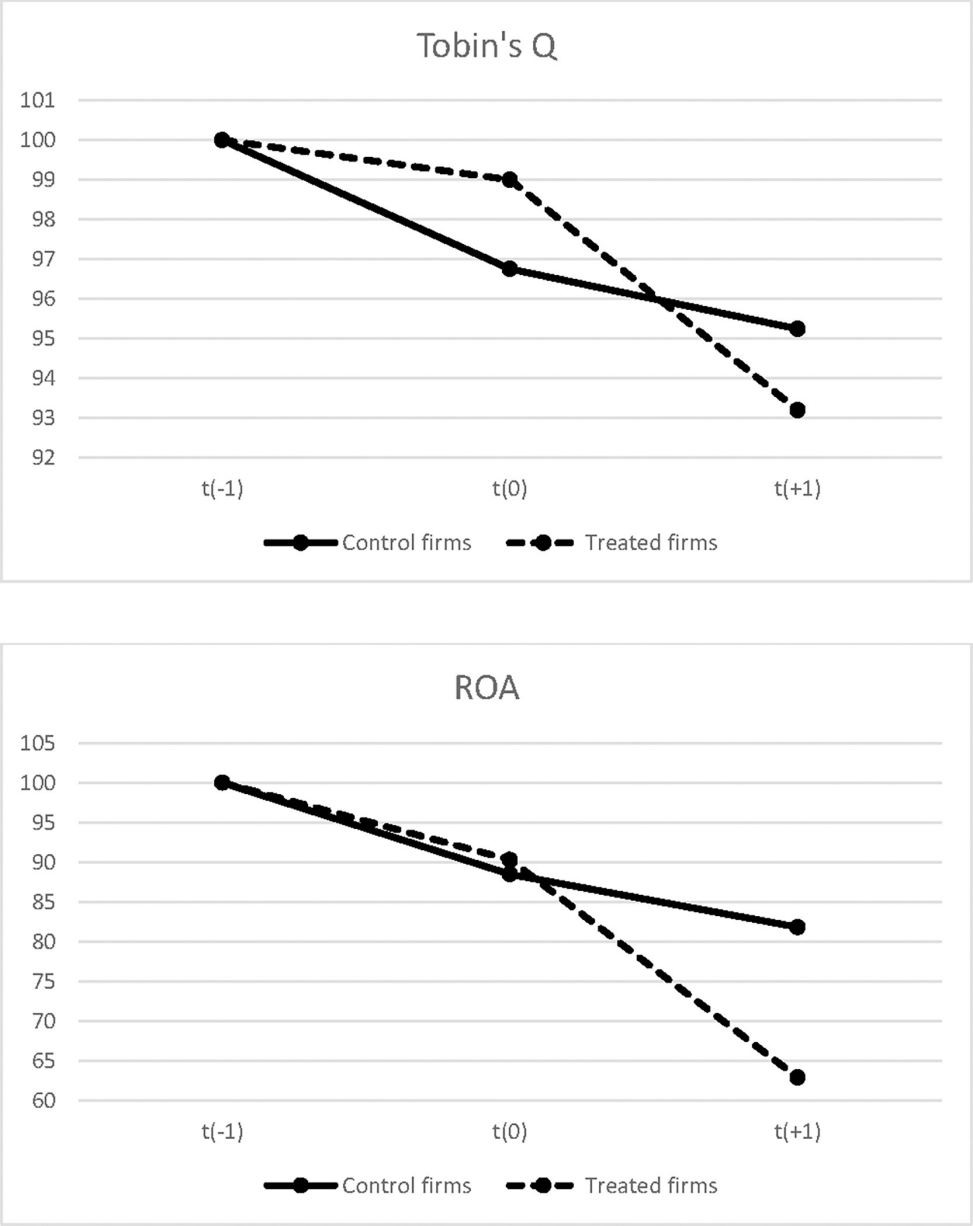
Performance Trends for firms exposed vs not exposed to flooding. This figure plots the average *Tobin’s Q* and *ROA* values in the year prior to the flooding year (i.e., year *t(-1)*), the flooding year (i.e., year *t(0)*) and the year after the flooding year (i.e., year *t(+1)*) for both the treated firms that experience an increase in flood ratio in year *t(0)* and the control firms that do not experience any flooding during these three years. The levels of *Tobin’s Q* and *ROA* in year *t(−1)* are rescaled to 100 for the ease of comparison between the two groups.

We further plot the average *Tobin’s Q* and *ROA* values in the year prior to the flooding year (i.e., year *t(-1)*), the flooding year (i.e., year *t(0)*) and the year after the flooding year (i.e., year *t(+1)*) for both the treated firms that experience an increase in flood ratio in year *t(0)* and the control firms that do not experience any flooding during these three years. The levels of *Tobin’s Q* and *ROA* in year *t(−1)* are rescaled to 100 for the ease of comparison between the two groups. As shown in Fig 2, we find that compared with the control firms, the treated firms experience a clear post-flooding reduction in both *Tobin’s Q* and *ROA* in year *t(+1)*, which is consistent with the baseline regression results in Table 2.

A potential concern is that if many firms in our sample are headquartered in a few major hub cities such as Beijing, Shanghai, Shenzhen and Guangzhou, one flood in a certain hub city can result in many repeated firm-year observations and potentially lead to an overestimation of the impact of flooding on firm performance. As a robustness check, we exclude all firms headquartered in Beijing, Shanghai, Shenzhen and Guangzhou from the sample and reestimate the performance regressions. As shown in Table A2 in the S1 Appendix, the regression results are qualitatively very similar to those presented in Table 2. In fact, the economic magnitudes of the effects of flooding on the performance measures are also similar across the two tables.

### Addressing the endogeneity concerns on the baseline results

Our key independent variable, *Floodratio*, is arguably exogenous to the firm and, thus, controlling for firm fixed effects, year fixed effects, firm and managerial characteristics and city-level economic development (at the yearly level) should be sufficient to identify the impact of flooding on future firm performance. However, there can still be a concern that the adverse effect of flooding on firm performance may be driven by poor public choices and/or inefficient crisis management of firms’ headquarter cities, which could be associated with both severe flooding and poor firm performance. Moreover, unobservable local economic dynamics (e.g., the development of a city’s infrastructure) can also be related to both the severity of flooding and firm performance, and the flood ratio measure can be subject to measurement errors.

To investigate whether local economic conditions and crisis management abilities affect our baseline results, in each year we match treatment firms (i.e., flood-exposed firms) with control firms (i.e., firms not exposed to flooding) that are in the same industry as treatment firms and located very close to treatment firms to form a matched sample. The rationale of this identification strategy is that firms located very close to each other should face very similar local economic conditions and local crisis management abilities. We then reestimate the baseline regression results using this matched sample.

Specifically, when constructing the matched sample, we first define treatment firms as those firms exposed to flooding in a year during our sample period. We then match each treatment firm with a control firm that are not exposed to flooding in that particular year and are located in the same province, operating in the same industry (2-digit industry classification by the China Securities Regulatory Commission), and having the closest total assets to the treatment firm. We also require the matched pair of treatment and control firms to be located very close to each other, with the geographic distance being less than 100 kilometres or 50 kilometres, so that they are likely to face very similar local economic conditions and crisis management abilities.

The regression results using this matched sample are reported in Table 3. In Panel A, we require the geographic distance between the matched treatment and control firms to be less than 100 kilometres. The regression results consistently show that the estimated coefficients of *Floodratio* remain negative and statistically significant across different regressions using different firm performance measures as dependent variables. Moreover, the magnitudes of the coefficient estimates of *Floodratio* using the matched sample are comparable or even larger than the magnitudes of those reported in Table 2 using the full sample. The regression results are qualitatively similar in Panel B when we further restrict the geographic distance to be less than 50 kilometres. These results further confirm that our finding of a significant, negative impact of flooding on firm performance is most likely causal and not driven by unobservable local economic conditions or crisis management abilities.

**Table 3:** Regression results using matched samples. This table reports the results on the effects of flooding on firm performance using the matched sample. To construct the matched sample, we first define treatment firms as firms exposed to flooding in a year during our sample period. We then match each treatment firm with a control firm that is not exposed to flooding in that year and is located in the same province, operating in the same industry (2-digit industry classification by the China Securities Regulatory Commission), and having the closest total assets as the treatment firm. We also require the matched pair of treatment and control firms to be located very close to each other, with the geographic distance being less than 100 kilometres or 50 kilometres, so that they are likely to face very similar local economic conditions and crisis management abilities. In Panel A, we require the geographic distance between the matched treatment and control firms to be less than 100 kilometres. In Panel B, we further restrict the geographic distance to be less than 50 kilometres. Control variables used in Table 2 are included in columns 2 and 4. Variable definitions are in Table A1 in the S1 Appendix. Robust standard errors are clustered at the city-year level and reported in parentheses. *, ** and *** indicate the significance levels at 10%, 5% and 1%, respectively.

Dependent	(1)	(2)	(3)	(4)
Variables	Tobin’s Q		ROA	
Panel A: Geographic distance < 100 kilometres				
Floodratio	-1.546**	-0.910*	-0.064*	-0.065*
	(0.758)	(0.483)	(0.034)	(0.038)
Observations	15,069	15,069	14,223	14,223
Adjusted R-squared	0.596	0.734	0.315	0.467
Control variables	No	Yes	No	Yes
Year fixed effects	Yes	Yes	Yes	Yes
Firm fixed effects	Yes	Yes	Yes	Yes
Panel B: Geographic distance < 50 kilometres				
Floodratio	-2,749**	-1.086	-0.151***	-0.135***
	(1.052)	(0.741)	(0.056)	(0.048)
Observations	3,846	3,846	3,882	3,882
Adjusted R-squared	0.556	0.736	0.377	0.591
Control variables	No	Yes	No	Yes
Year fixed effects	Yes	Yes	Yes	Yes
Firm fixed effects	Yes	Yes	Yes	Yes

We further employ the instrumental-variable regression approach developed by our colleague [5] to address the potential endogeneity concerns and identify the impact of flooding on firm performance. This approach does not reply on any external instrument, but instead exploits the heterogeneity in the error term of the first stage regression to generate instrument from within the existing model. This method has also been applied in recent corporate finance research [29–31]. The results are reported in Table A3 in the S1 Appendix. We find that the coefficient estimates of the instrumented flood ratio using this estimation method continue to be negative and significant for both the *Tobin’s Q* and *ROA* regressions.

**Fig 3:**
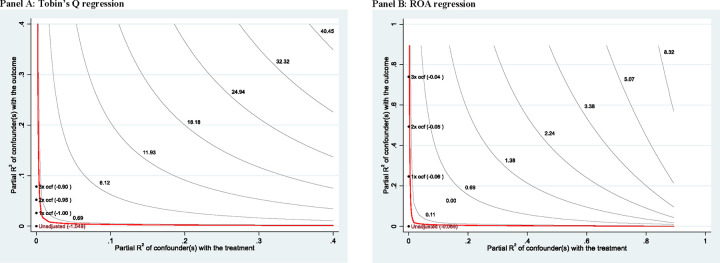
Sensitivity of OLS estimates to omitted variables. This figure shows how sensitive the OLS estimates are to omitted variables using the approach of Cinelli and Hazlett (2020). The figure plots the adjusted point estimates of the efficient of *Floodratio* (our main variable of interest), when including an unobservable variable in the regression. The horizontal axis describes the fraction of the residual variation in *Floodratio* explained by the confounder, and the vertical axis describes the fraction of the residual variation in *Tobin’s Q* or *ROA* explained by the confounder (Panel A for the *Tobin’s Q* regression and Panel B for the *ROA* regression). We use *OCF* (i.e., Operating cash flows / Total assets) as the benchmark variable. *OCF* is highly signficantly and positively correlated with both *Tobin’s Q* and *ROA* at the 1% level (the correlation coefficient is 0.189 between *OCF* and *Tobin’s Q* and 0.527 between *OCF* and *ROA*). In Panel A (for the *Tobin’Q* regression) and Panel B (for the *ROA* regression), the three points on each plot show the bounds on the partial R^2^ of the unobserved confounder if it were k (k = 1, 2 or 3) times “as strong” as the benchmark variable *OCF*. Both plots show that the signs of the point estimates of the efficient of *Floodratio* are still negative and relatively insensitive to a confounder with various strengths of the benchmark variable.

We also examine how sensitive the OLS estimates are to omitted variables using the approach of our colleagues [4]. Fig 3 plots the adjusted point estimates of the efficient of *Floodratio* when including an unobservable variable in the regression. The horizontal axis describes the fraction of the residual variation in *Floodratio* explained by the confounder, and the vertical axis describes the fraction of the residual variation in *Tobin’s Q* or *ROA* explained by the confounder (Panel A for the *Tobin’s Q* regression and Panel B for the *ROA* regression). We use *OCF* (i.e., Operating cash flows / Total assets) as the benchmark variable. *OCF* is highly signficantly and positively correlated with both *Tobin’s Q* and *ROA* at the 1% level (the correlation coefficient is 0.189 between *OCF* and *Tobin’s Q* and 0.527 between *OCF* and *ROA*). In Panel A (for the *Tobin’Q* regression), the three points on the plot show the bounds on the partial R^2^ of the unobserved confounder if it were k (k = 1, 2 or 3) times “as strong” as the benchmark variable *OCF*. We find that the signs of the point estimates of the efficient of *Floodratio* are still negative and relatively insensitive to a confounder with various strengths of our benchmark variable. The finding is similar in Panel B (for the *ROA* regression).

### Sudden flooding vs normal flooding

Some floods may be more predictable than others. For example, in some cities flooding can happen around a specific time every year. Thus, local firms in those cities are prepared for the occurrence of such expected flooding, which should result in less severe impact on firm performance. However, if flooding is sudden or unexpectedly severe in a city-year, it should impose a stronger impact on firm performance.

We thus examine whether sudden floods and expected floods have differential impacts on corporate performance by splitting our main variable of interest, flood ratio (*Floodratio*) into two variables *Floodratio_sudden and Floodratio_normal*. *Floodratio_sudden* equals *Floodratio* if the value of *Floodratio* of a city in a year is at least two-standard-deviation larger than the average *Floodratio* of that city (where the firm’s headquarter is located) during our sample period and equals 0 otherwise. Similarly, *Floodratio_normal* equals *Floodratio* if the value of *Floodratio* of a city in a year is *not* at least two-standard-deviation larger than the average *Floodratio* of that city during our sample period and equals 0 otherwise. As shown in Table 4, we find that the impact of flooding on future firm performance is mainly driven by sudden floods and not by expected floods. These results again support the causal interpretation of the negative impact of flooding on future firm performance. In Table A4 in the S1 Appendix, we also investigate the effects of flooding on two alternative measures of future firm performance, one-year-ahead future stock returns (Stock returns) and return on sales (ROS), and document qualitatively similar results.

**Table 4:** Sudden flooding vs expected flooding. This table reports the results of the effects of both sudden flooding and expected flooding on firm performance using various proxies for firm performance, including *Tobin’s Q* and *ROA*, which are measured in year *t+*1. We split our main variable of interest, flood ratio (*Floodratio*) into two variables *Floodratio_sudden and Floodratio_normal*. *Floodratio_sudden* equals *Floodratio* if the value of *Floodratio* of a city in a year is at least two-standard-deviation larger than the average *Floodratio* of that city (where the firm’s headquarter is located) during our sample period and equals 0 otherwise. Similarly, *Floodratio_normal* equals *Floodratio* if the value of *Floodratio* of a city in a year is *not* at least two-standard-deviation larger than the average *Floodratio* of that city during our sample period and equals 0 otherwise. All independent variables are measured in year *t*. Variable definitions are in Table A1 in the S1 Appendix. Robust standard errors are clustered at the city-year level and reported in parentheses. *, ** and *** indicate the significance levels at 10%, 5% and 1%, respectively.

Dependent	(1)	(2)	(3)	(4)
Variables	Tobin’s Q		ROA	
Floodratio_sudden	-1.422***	-0.783**	-0.050*	-0.054**
	(0.571)	(0.383)	(0.032)	(0.027)
Floodratio_normal	2.873	-3.342	1.131	0.469
	(10.206)	(8.370)	(0.473)	(0.454)
Size		-0.842***		-0.013***
		(0.056)		(0.001)
Leverage		0.579***		0.014***
		(0.088)		(0.005)
Largest		0.136		0.052***
		(0.091)		(0.006)
Ln(board)		0.128**		-0.002
		(0.064)		(0.004)
Indep		0.186		0.007
		(0.178)		(0.010)
SOE		-0.119***		-0.003
		(0.042)		(0.003)
OCF		3.203***		0.281***
		(0.307)		(0.013)
Tangibility		-0.246***		0.014***
		(0.081)		(0.005)
Cash holding		0.526***		0.063***
		(0.110)		(0.005)
CEO age		0.007		0.001
		(0.069)		(0.004)
CEO tenure		-0.055***		0.000
		(0.012)		(0.001)
Volatility		2.449***		0.014**
		(0.405)		(0.006)
Market-to-book		0.125*		0.000
		(0.070)		(0.001)
Past stock returns		0.219***		0.003***
		(0.028)		(0.001)
GDP		-0.169		-0.005
		(0.333)		(0.013)
Income per capita		0.443		0.011
		(0.281)		(0.009)
Past GDP growth		-0.234		-0.004
		(0.240)		(0.008)
Observations	29,808	29,808	29,635	29,635
Adjusted R-squared	0.582	0.728	0.300	0.443
Year fixed effects	Yes	Yes	Yes	Yes
Firm fixed effects	Yes	Yes	Yes	Yes

In summary, the empirical results from various identification strategies suggest that the negative impact of flooding on future firm performance is most likely causal.

### Subsample analyses

Next, we conduct a set of subsample analyses to study settings in which the effects of flooding on firm performance are expected to vary. First, compared with intangible knowledge assets, tangible capital assets such as plants, equipment and warehouses are more likely to be damaged by flooding. Thus, we expect the negative impact of flooding on future firm performance to be more pronounced for firms with more intensive tangible asset investment.

Second, existing studies suggest that high-quality governments are better able to provide high-quality infrastructures and public services than low-quality ones [32, 33]. High-quality governments may also have more efficient crisis management systems in place, which can help mitigate the negative impact of flooding on firm performance. We thus expect the performance of firms located in those cities with high government quality to be less affected by flooding.

Third, compared with financially unconstrained firms, financially constrained firms do not have enough financial resources to absorb the negative shocks occasioned by flooding. Thus, flooding can more severely disrupt their business operations and limit their investment opportunities. We hence expect the effect of flooding on firm performance to be stronger for financially constrained firms than for financially unconstrained firms.

Fourth, compared to non-SOEs, SOEs are more likely to receive various government financial supports such as government subsidies and cheap bank loans [7, 8]. Thus, SOEs, relative to non-SOEs, should be better able to cushion the negative flood shocks due to government supports. Finally, although firms may choose to locate in an area more likely to be affected by flooding for a reason (e.g., locate in a city close to a river for logistics concern), those firms that spread their business operations across many cities should be able to mitigate the negative impact of flooding on their overall performance. We thus expect the negative effect of flooding on firm performance to be weaker for geographically diversified firms.

We sort firms into subsamples by tangible capital expenditure (above vs below the sample median *CAPEX* in each year), government quality (above vs below the sample median marketization index), financial constraints (above vs below the sample median Hadlock and Pierce’s (2010) size-age index in each year), state ownership status (i.e., whether the ultimate owner is the government), and operation dispersion (above vs below the sample median number of cities that the firm has operations in each year), respectively. Note that the marketization index is developed by our colleagues [34]. This index measures the overall market development in China and consists of 5 sub-indexes, namely (1) government-market relationship; (2) development of non-state capitalism; (3) development of product market; (4) development of factor market; and (5) development of financial intermediaries and law institutions. In this study, we use the government-market relationship sub-index to measure government quality—a higher value indicates the local government is generally more market-oriented and thus more efficient.

We then estimate Eq (1) using each subsample separately. Control variables, firm fixed effects and year fixed effects are included in all subsample regressions. We further conduct Chow tests to test the *Floodratio* coefficient differences between subsamples. Table 5 reports the estimation results. For brevity, we only report the coefficient estimates of *Floodratio* for the subsamples and the *p*-values of Chow tests for the coefficient differences between subsamples.

**Table 5:** Cross-sectional analysis using subsamples. This table reports the subsample regression results of the effects of flooding on firm performance. The sample firms are sorted by capital expenditure (above vs below the sample median *CAPEX* in each year) in Panel A, by marketization index (above vs below the sample median marketization index) in Panel B, by financial constraints (above vs below the sample median SA index in each year) in Panel C, by state ownership status (non-SOEs vs SOEs) in Panel D, and by operation dispersion (above vs below the sample median number of operating cities in each year) in Panel E, respectively. Control variables, firm fixed effects and year fixed effects are included in all subsample regressions. Chow tests are conducted to formally test the *Floodratio* coefficient differences between subsamples. Variable definitions are in Table A1 in the S1 Appendix. Robust standard errors are clustered at the city-year level and reported in parentheses. *, ** and *** indicate the significance levels at 10%, 5% and 1%, respectively.

Dependent	(1)	(2)
Variables	Tobin’s Q	ROA
*Panel A*: *High tangibility vs low tangibility*		
Floodratio	-0.659*	-0.073***
(High tangibility, n = 14,904)	(0.362)	(0.027)
Floodratio	-0.208	0.021
(Low tangibility, n = 14,904)	(0.603)	(0.028)
P-value of Chow test (High—Low)	0.113	0.047
*Panel B*: *High marketization index vs low marketization index)*		
Floodratio	-0.051	-0.042
(High quality, n = 14,101)	(0.528)	(0.030)
Floodratio	-1.304**	-0.065*
(Low quality, n = 15,707)	(0.572)	(0.040)
P-value of Chow test (High—Low)	0.003	0.078
*Panel C*: *High financial constraints vs low financial constraints*		
Floodratio	-0.985*	-0.059*
(High constraints, n = 14,904)	(0.527)	(0.043)
Floodratio	-0.072	-0.030
(Low constraints, n = 14,904)	(0.253)	(0.020)
P-value of Chow test (High—Low)	0.012	0.097
*Panel D*: *SOEs vs non-SOEs*		
Floodratio	-1.481**	-0.056**
(Non-SOEs, n = 16,103)	(0.593)	(0.028)
Floodratio	-0.317	-0.062
(SOEs, n = 13,705)	(0.322)	(0.042)
P-value of Chow test (Non-SOEs—SOEs)	0.067	0.134
*Panel E*: *Large subsidiary number vs small subsidiary number*		
Floodratio	-0.194	0.004
(Large number, n = 13,523)	(0.278)	(0.039)
Floodratio	-1.327**	-0.117***
(Small number, n = 14,649)	(0.645)	(0.035)
P-value of Chow test (Large–Small)	0.023	0.007

Consistent with our conjectures, we find that the negative impact of flooding on one-year-ahead firm performance is significantly stronger for firms with more intensive tangible asset investment, firms headquartered in cities with low-quality governments, financially constrained firms, non-SOEs, and firms with low geographic dispersion. The coefficient estimates of *Floodratio* are negative and statistically significant across different performance regressions for firms with high tangible asset investment, firms headquartered in cities with low-quality governments, financially constrained firms, non-SOEs, and geographically concentrated firms. In contrast, the coefficient estimates are generally negative but statistically insignificant for firms with low tangible asset investment, firms headquartered in cities with high-quality governments, financially unconstrained firms, SOEs and geographically dispersed firms. The coefficient differences between subsamples are also generally statistically significant (with two exceptions). The results from these subsample analyses further support the causal interpretation of the effects of flooding on future firm performance.

### Firms with versus without insurance compensation

Although the earlier findings suggest that flooding negatively impacts firm performance, we conjecture that some firms may receive insurance compensation which can (partially) cushion the flood impact. Thus, we further separate our regression sample into two subsamples based on whether a firm receives insurance payment in a year and reestimate the performance regressions. Information on insurance payment is obtained from the CSMAR database (unfortunately, the reason for receiving insurance payment is often not specified). As shown in Table A5 in the S1 Appendix, we indeed find that the negative impact of flooding on firm performance mostly concentrates in firms without insurance compensation.

### Results on contemporaneous and long-term future performance

We further examine whether flooding influences firms’ contemporaneous and long-term future performance. We reestimate Eq (1) using the firm performance variables measured in year *t* (i.e., the same year as the flood ratio measurement year), year *t+*2 (i.e., the second year after the flood ratio measurement year) or year *t+*3 (i.e., the third year after the flood ratio measurement year) as the dependent variables. As shown in Table A6 in the S1 Appendix, the coefficient estimates of *Floodratio* are statistically insignificant across different regressions. These results suggest that the impact of flooding on firm performance is relatively short run in nature and mainly reflected in the year after flooding.

## The effects of flooding on firm policies

Next, we turn to firm policy reactions and investigate the effects of flooding on exposed firms’ investment, financial, cash, payout and executive compensation policies. It is worthy to note that although we follow the literature to include control variables in the firm policy regressions, the findings on the impact of flooding on firm policies do not change qualitatively when we exclude the control variables from the regressions (i.e., when we only control for firm and year fixed effects in the firm policy regressions).

### The effects of flooding on firms’ investment policies

To investigate the effects of flooding on firms’ investment policies, we use the following regression specification:

$${Investment}_{ict+1}=\alpha_{1}{Floodratio}_{ct}+\alpha_{2}{Size}_{ict}+\alpha_{3}{Leverage}_{ict}{{+\alpha }_{4}{Largest}_{ict}+\alpha_{5}Lnboard}_{ict}+{\alpha_{6}{Indep}_{ict}+\alpha_{7}SOE}_{ict}{{+\alpha }_{8}{OCF}_{ict}+\alpha_{9}Tobin^{\prime }s\;Q}_{ict}+{\alpha_{10}CEO\;age}_{ict}+\alpha_{11}{CEO\;tenure}_{ict}{+\alpha_{12}Past\;stock\;returns}_{ict}+\alpha_{13}{GDP}_{ct}+{\alpha_{14}Income\;per\;capita}_{ct}+\alpha_{15}{GDP\;growth}_{ct}+{Firm}_{i}+{Year}_{t}+\varepsilon_{it}$$ (2)

In Eq (2), *Investment* is a measure of firms’ investment policies in year *t+*1. We construct two investment variables, *R&D* and *CAPEX*, to measure the firm’s intangible assets investments and tangible assets investments, respectively. *R&D* is defined as the ratio of R&D expenditures to total assets, and *CAPEX* is defined as the ratio of capital expenditures in property, plant and equipment to total assets. We also consider firms’ mergers and acquisitions (M&A) activities and construct two M&A-related variables: *MA* is an indicator variable which equals 1 if the firm announces M&A transaction(s) in a year (which is eventually completed) and equals 0 otherwise; *MA_exp* is the ratio of expenses on M&A to total assets of the firm. We further distinguish the locations of target firms and construct two additional variables: *MA_nonflood* is an indicator variable which equals 1 if the firm announces M&A in a year and the target firm’s headquarter city is not affected by flooding in that year and equals 0 otherwise; *MA_flood* is an indicator variable which equals 1 if the firm announces M&A and the target firm’s headquarter city is affected by flooding and equals 0 otherwise. We include a set of control variables (which are defined earlier) following prior studies [35, 36]. Both firm and year fixed effects are included when estimating Eq (2). Standard errors are clustered at the city-year level.

Table 6 reports the results with regards to firm investment policies. First, columns 1 and 2 of Table 6 report the results using the expenditures on intangible knowledge assets (*R&D*) and tangible capital assets (*CAPEX*) as the dependent variables, respectively. The sample size of the R&D regression is smaller due to missing data on R&D expenditures for some firm-year observations. The results show that the coefficient estimate of *Floodratio* is positive and statistically significant at the 5% level in column 1, and negative and statistically significant at the 10% level in column 2. Although intangible knowledge assets and tangible capital assets can both increase firm productivity, the latter is far more vulnerable to the threat of flooding than the former. Our finding that flood-exposed firms increase their expenditures on R&D but decrease their expenditures on tangible capital assets thus indicates that such firms substituting intangible assets for tangible assets in production to mitigate the impact of flooding. In terms of economic magnitudes, a one-standard-deviation increase in *Floodratio* is associated with an increase of 1.08% in one-year-ahead R&D expenditures and a decrease of 15.14% in one-year-ahead capital assets expenditures, relative to their respective sample medians.

**Table 6:** Flooding and firm investment policy. This table reports the regression results of the effects of flooding on firm investment policy. Dependent variables are R&D expenditures to total assets (*R&D*) in column 1, capital expenditures to total assets (*CAPEX*) in column 2, probability of conducting M&As (*MA*) in column 3, M&A expenses to total assets (*MA_exp*) in column 4, probability of conducting M&As in non-flooding city (*MA_nonflood*) in column 5, and probability of conducting M&As in flooding city (*MA_flood*) in column 6, respectively. Variable definitions are in Table A1 in the S1 Appendix. Robust standard errors are clustered at the city-year level and reported in parentheses. *, ** and *** indicate the significance levels at 10%, 5% and 1%, respectively.

Dependent	(1)	(2)	(3)	(4)	(5)	(6)
Variables	R&D	CAPEX	MA	MA_exp	MA_nonflood	MA_flood
Floodratio	0.013**	-0.424*	0.410**	0.033**	0.277*	0.133
	(0.006)	(0.293)	(0.183)	(0.015)	(0.173)	(0.120)
Size	-0.004***	0.020***	-0.039***	-0.003***	-0.023***	-0.016***
	(0.000)	(0.007)	(0.008)	(0.001)	(0.007)	(0.006)
Leverage	0.000	-0.045	-0.035	-0.003	-0.006	-0.029
	(0.001)	(0.042)	(0.030)	(0.002)	(0.024)	(0.021)
Largest	-0.000	0.093	0.118***	0.009***	0.064*	0.054*
	(0.002)	(0.088)	(0.041)	(0.003)	(0.036)	(0.032)
Lnboard	0.002*	-0.047	-0.010	-0.001	0.023	-0.033
	(0.001)	(0.042)	(0.031)	(0.002)	(0.027)	(0.023)
Indep	-0.000	0.067	-0.030	-0.002	0.030	-0.060
	(0.003)	(0.098)	(0.082)	(0.007)	(0.069)	(0.064)
SOE	0.000	0.010	-0.029	-0.002	-0.013	-0.015
	(0.001)	(0.033)	(0.019)	(0.002)	(0.016)	(0.014)
OCF	0.005**	0.222**	0.205***	0.016***	0.084	0.121**
	(0.002)	(0.093)	(0.071)	(0.006)	(0.060)	(0.053)
Tobin’s Q	0.001***	0.009*	0.004	0.000	0.003	0.001
	(0.000)	(0.005)	(0.003)	(0.000)	(0.002)	(0.002)
CEO_age	0.001	0.054*	-0.030	-0.002	-0.023	-0.006
	(0.001)	(0.032)	(0.031)	(0.003)	(0.026)	(0.024)
CEO_tenure	0.000***	-0.017**	-0.003	-0.000	-0.000	-0.003
	(0.000)	(0.008)	(0.006)	(0.000)	(0.005)	(0.004)
Past stock returns	0.000	-0.001	0.017***	0.001***	0.013***	0.004
	(0.000)	(0.005)	(0.005)	(0.000)	(0.004)	(0.004)
GDP	0.012*	-0.024	-0.141	-0.011	-0.138	-0.004
	(0.007)	(0.081)	(0.099)	(0.008)	(0.086)	(0.084)
Income per capita	-0.011	0.031	0.066	0.005	0.057	0.009
	(0.007)	(0.072)	(0.076)	(0.006)	(0.063)	(0.067)
Past GDP growth	0.010***	0.014	0.063	0.005	0.110*	-0.048
	(0.003)	(0.066)	(0.078)	(0.006)	(0.063)	(0.056)
Observations	16,331	27,385	25,115	25,115	25,115	25,115
Adjusted R-squared	0.833	0.132	0.231	0.231	0.190	0.191
Year fixed effects	Yes	Yes	Yes	Yes	Yes	Yes
Firm fixed effects	Yes	Yes	Yes	Yes	Yes	Yes

In addition, we examine firms’ M&A activities and report the results in columns 3 to 6. The dependent variable in column 3 is whether firms conduct M&A in a year and the dependent variable in column 4 is the amount of M&A expenditures scaled by total assets in a year. Interestingly, the coefficient estimate of *Floodratio* is positive and statistically significant at the 5% level in both regressions, indicating that flood-exposed firms are more likely to announce M&A deals and spend more on M&A transactions. In terms of economic magnitude, a one-standard-deviation increase in *Floodratio* is on average associated with an increase of 2.11% in one-year-ahead acquisition likelihood and an increase of 2.15% in one-year-ahead M&A expenditures, relative to their respective sample means (note that the sample medians of both *MA* and *MA_exp* are 0).

Lastly, the results in columns 5 and 6 show that flood-exposed firms are more likely to announce acquisitions of target firms located in cities unaffected by flooding, but not more likely to acquire target firms located in flood-affected cities. This finding suggests that flood-affected firms try to acquire targets located in non-flooded areas and shift their operations to the target firms to mitigate the negative impact of flooding.

### The effects of flooding on firms’ financing and cash policies

To investigate the effects of flooding on firms’ financing and cash policies, we use the following regression specification:

$${Financing}_{ict+1}=\alpha_{1}{Floodratio}_{ct}+\alpha_{2}{Size}_{ict}{+\alpha_{3}{Leverage}_{ict}+\alpha_{4}{Largest}_{ict}+\alpha_{5}Lnboard}_{ict}+{\alpha_{6}{Indep}_{ict}+\alpha_{7}SOE}_{ict}{{+\alpha }_{8}{OCF}_{ict}+\alpha_{9}ROS}_{ict}+\alpha_{10}{Sales\;growth}_{ict}{+\alpha_{11}{Dividend}_{ict}+\alpha_{12}CEO\;age}_{ict}+\alpha_{13}{CEO\;tenure}_{ict}{+\alpha_{14}Past\;stock\;returns}_{ict}+{\alpha_{15}{GDP}_{ct}+\alpha_{16}Income\;per\;capita}_{ct}+\alpha_{17}{GDP\;growth}_{ct}+{Firm}_{i}+{Year}_{t}+\varepsilon_{it}$$ (3)

In Eq (3), *Financing* represents the proxies for firms’ financing and cash holding policies. We consider three aspects of firms’ financing and cash holding policies. The first aspect is firm leverage level. We construct two leverage variables: *Book leverage* is the ratio of the firm’s book value of total debt to book value of total assets; *Market leverage* is the ratio of the firm’s book value of total debt to the sum of book value of total debt and market value of equity. The second aspect is the firm’s debt maturity. We construct a variable *Debt maturity*, which is defined as the ratio of long-term debt to total debt. The third aspect is the firm’s cash holding. We measure *Cash holding* as the ratio of cash and cash equivalents to total assets.

We follow the extant literature [37] to include relevant control variables. *Dividend* is an indicator variable that equals 1 if a firm pays cash dividends in the year and equals 0 otherwise. Other variables are defined earlier. Both firm and year fixed effects are included when estimating Eq (3).

Table 7 reports the regression results on firms’ financing and cash holding policies. In columns 1 and 2, the dependent variable is *Book leverage* and *Market leverage*, respectively. In column 3, the dependent variable is *Debt maturity*. The estimated coefficients of *Floodratio* are positive in columns 1 to 3 and statistically significant (at the 5% level) in columns 1 and 3, indicating that flood-exposed firms tend to increase their leverage and debt maturity. In terms of economic magnitudes, a one-standard-deviation increase in *Floodratio* is on average associated with 0.32% increase in *Book leverage* and 6.30% increase in *Debt maturity*, relative to their respective sample medians. In column 4, the dependent variable is *Cash holding*. We find that the coefficient estimate of *Floodratio* is positive and statistically significant at the 5% level, indicating that flood-affected firms tend to increase their cash holdings to maintain sufficient cash reserve for pre-cautionary motive. A one-standard-deviation increase in *Floodratio* is associated with a 0.75% increase in cash holding relative to its sample median.

**Table 7:** Flooding and firm financing policy. This table reports the regression results of the effects of flooding on firm financing policy. Dependent variables are book leverage (*Book leverage*) in column 1, market leverage (*Market leverage*) in column 2, debt maturity (*Debt Maturity*) in column 3, and cash holding levels (*Cash holding*) in column 4, respectively. Variable definitions are in Table A1 in the S1 Appendix. Robust standard errors are clustered at the city-year level and reported in parentheses. *, ** and *** indicate the significance levels at 10%, 5% and 1%, respectively.

Dependent	(1)	(2)	(3)	(4)
Variables	Book leverage	Market leverage	Debt maturity	Cash holding
Floodratio	0.097**	0.056	0.084**	0.063**
	(0.045)	(0.040)	(0.038)	(0.030)
Size	0.067***	0.112***	0.044***	-0.019***
	(0.002)	(0.002)	(0.002)	(0.001)
Leverage				-0.108***
				(0.007)
Largest	-0.027**	0.012	0.045***	0.034***
	(0.012)	(0.010)	(0.011)	(0.008)
Lnboard	0.000	-0.030***	-0.014**	0.009
	(0.008)	(0.007)	(0.007)	(0.006)
Indep	-0.010	0.021	0.017	0.033**
	(0.022)	(0.019)	(0.019)	(0.015)
SOE	0.003	0.021***	-0.006	-0.001
	(0.006)	(0.004)	(0.004)	(0.004)
OCF	-0.477***	-0.566***	-0.147***	0.161***
	(0.031)	(0.025)	(0.027)	(0.020)
ROS	-0.083***	-0.023***	0.036***	-0.006
	(0.012)	(0.008)	(0.012)	(0.007)
Sales growth	0.011***	-0.001	0.003**	0.003**
	(0.002)	(0.001)	(0.002)	(0.001)
Dividend	-0.014***	-0.023***	0.001	0.011***
	(0.002)	(0.002)	(0.002)	(0.002)
CEO_age	-0.000	-0.015**	0.005	0.001
	(0.009)	(0.007)	(0.007)	(0.006)
CEO_tenure	0.002	0.002**	-0.001	-0.005***
	(0.001)	(0.001)	(0.001)	(0.001)
Past stock returns	0.006***	-0.014***	0.003***	-0.004***
	(0.001)	(0.001)	(0.001)	(0.001)
GDP	0.010	0.052*	-0.025	0.002
	(0.030)	(0.027)	(0.027)	(0.023)
Income per capita	-0.014	-0.078***	-0.016	-0.007
	(0.024)	(0.022)	(0.021)	(0.019)
Past GDP growth	0.197***	0.023	0.065***	-0.206***
	(0.024)	(0.019)	(0.020)	(0.023)
Observations	27,125	28,822	28,822	27,109
Adjusted R-squared	0.775	0.825	0.615	0.594
Year fixed effects	Yes	Yes	Yes	Yes
Firm fixed effects	Yes	Yes	Yes	Yes

### The effects of flooding on firms’ payout policies

To investigate the effects of flooding on firms’ payout policies, we use the following regression specification:

$${Payout}_{ict+1}=\alpha_{1}{Floodratio}_{ct}+\alpha_{2}{Size}_{ict}{+\alpha_{3}{Leverage}_{ict}+\alpha }_{4}{Largest}_{ict}+\alpha_{5}{Lnboard}_{ict}+\alpha_{6}{Indep}_{ict}+\alpha_{7}{SOE}_{ict}{{+\alpha }_{8}{OCF}_{ict}+\alpha_{9}ROS}_{ict}+\alpha_{10}{Sales\;Growth}_{ict}{+\alpha_{11}{Dividend}_{ict}+\alpha_{12}CEO\;age}_{ict}+\alpha_{13}{CEO\;tenure}_{ict}{+\alpha_{14}Past\;stock\;returns}_{ict}+\alpha_{15}{GDP}_{ct}+\alpha_{16}{Income\;per\;capita}_{ct}+\alpha_{17}{GDP\;growth}_{ct}+{Firm}_{i}+{Year}_{t}+\varepsilon_{it}$$ (4)

In Eq (4), *Payout* represents the proxies for firms’ payout policies. We consider both cash dividend policy and share repurchase policy. To measure cash dividend policy, we construct three variables: *Dividend* is an indicator variable which equals 1 if the firm pays cash dividends in a year and equals 0 otherwise; *DPS* is dividend per share, defined as the ratio of cash dividend payment to total shares outstanding; *DPS/Book per share* is the ratio of cash dividend per share to book value of equity per share. To measure repurchase policy, we employ two variables: *Repurchase ratio* is defined as the repurchased share value to market value of equity; *Repurchase* is an indicator variable that equals 1 if the firm buys back common shares outstanding in a year and equals 0 otherwise. Control variables follow the prior literature [38] and are defined earlier.

Table 8 reports the results on firms’ payout policies. In the first three columns, the dependent variable is the cash dividend payment dummy (*Dividend*), cash dividend per share (*DPS*) and the ratio of cash dividend per share to book value per share (*DPS/Book per share*), respectively. The results show that the coefficient estimates of *Floodratio* are negative in all three regressions and statistically significant in columns 1 and 3, indicating that flood-exposed firms tend to pay less cash dividend. In terms of economic magnitude, a one-standard-deviation increase in *Floodratio* is on average associated with a 1.03% decrease in the likelihood of paying cash dividend relative to the sample mean likelihood (note that the sample median of *Dividend* is 1) and a 18.23% decrease in the ratio of cash dividend per share to book value per share relative to the sample median ratio. In columns 4 and 5, the dependent variable is the share repurchase dummy (*Repurchase*) and the ratio of repurchase payment to market value of equity (*Repurchase ratio*), respectively. The results show that the coefficient estimates of *Floodratio* are positive and statistically significant at the 1% level in both regressions, indicating that flood-exposed firms are more likely to repurchase shares and have higher share repurchase ratio. In terms of economic magnitude, a one-standard-deviation increase in *Floodratio* is on average associated with a 0.26% increase in the ratio of repurchase payment to market value of equity relative to the sample mean ratio and a 102% increase in the likelihood of repurchasing common shares relative to the sample mean likelihood (note that the sample medians of both *Repurchase ratio* and *Repurchase* are 0).

**Table 8:** Flooding and firm payout policy. This table reports the regression results of the effects of flooding on firm payout policy. Dependent variables are the probability of paying cash dividends (*Dividend*) in column 1, dividend per share (*DPS*) in column 2, DPS to book value of equity per share (*DPS/Book per share*) in column 3, repurchase payment to market value of equity (*Repurchase ratio*) in column 4, and probability of repurchase (*Repurchase*) in column 5, respectively. Variable definitions are in Table A1 in the S1 Appendix. Robust standard errors are clustered at the city-year level and reported in parentheses. *, ** and *** indicate the significance levels at 10%, 5% and 1%, respectively.

Dependent	(1)	(2)	(3)	(4)	(5)
Variables	Dividend	DPS	DPS/Book per share	Repurchase ratio	Repurchase
Floodratio	-0.469**	-0.038	-0.158*	0.007***	5.712***
	(0.228)	(0.062)	(0.082)	(0.002)	(0.453)
Size	0.086***	0.020***	-0.001	0.000***	0.058***
	(0.006)	(0.004)	(0.001)	(0.000)	(0.004)
Leverage	-0.270***	-0.082***	-0.009*	-0.001***	-0.004
	(0.023)	(0.011)	(0.005)	(0.000)	(0.015)
Largest	0.166***	0.117***	0.021***	-0.001**	-0.167***
	(0.036)	(0.016)	(0.007)	(0.000)	(0.022)
Lnboard	0.035	-0.020	-0.001	0.000	0.012
	(0.024)	(0.014)	(0.005)	(0.000)	(0.014)
Indep	-0.079	0.276**	-0.001	-0.000	-0.048
	(0.067)	(0.120)	(0.010)	(0.000)	(0.036)
SOE	0.028**	-0.005	0.000	-0.000	-0.006
	(0.014)	(0.007)	(0.004)	(0.000)	(0.008)
OCF	1.842***	0.547***	0.204***	-0.001**	0.041
	(0.066)	(0.035)	(0.022)	(0.001)	(0.038)
Volatility	-0.024	0.037	0.070**	-0.001	-0.039
	(0.072)	(0.032)	(0.033)	(0.000)	(0.040)
Sales growth	-0.001	0.003	0.006***	-0.000	-0.012***
	(0.005)	(0.002)	(0.001)	(0.000)	(0.003)
ROS	0.067***	-0.046***	-0.019***	0.000	-0.024*
	(0.024)	(0.011)	(0.006)	(0.000)	(0.013)
Market-to-book	0.002***	0.001	-0.000	-0.000	0.002***
	(0.001)	(0.001)	(0.000)	(0.000)	(0.000)
CEO_age	0.023	0.035***	0.012***	0.000	0.011
	(0.024)	(0.009)	(0.004)	(0.000)	(0.015)
CEO_tenure	0.010**	-0.003	-0.003***	0.000***	0.016***
	(0.004)	(0.003)	(0.001)	(0.000)	(0.003)
Past stock returns	-0.012***	-0.003	-0.004***	-0.000**	0.002
	(0.004)	(0.003)	(0.001)	(0.000)	(0.002)
GDP	0.026	0.036	0.044**	0.000	0.162***
	(0.090)	(0.052)	(0.019)	(0.001)	(0.061)
Income per capita	-0.060	0.072*	-0.035**	-0.000	-0.126***
	(0.071)	(0.042)	(0.015)	(0.001)	(0.049)
Past GDP growth	-0.239***	-0.083*	-0.129***	0.000	0.208***
	(0.071)	(0.043)	(0.041)	(0.001)	(0.050)
Observations	28,808	27,146	26,753	28,808	28,808
Adjusted R-squared	0.528	0.575	0.222	0.186	0.432
Year fixed effects	Yes	Yes	Yes	Yes	Yes
Firm fixed effects	Yes	Yes	Yes	Yes	Yes

Given that cash dividend represents a commitment to shareholders [14] while share repurchase is more flexible [15–17], the results in Table 8 suggest that flood-exposed firms opt for paying less cash dividend and repurchasing more common shares outstanding to increase financial flexibility and mitigate the impact of flooding. We further examine the potential effects of flooding on firms’ long-term future investment, financing and payout policies. The generally insignificant results, shown in Table A7 in the S1 Appendix, suggest that flooding does not exert long-term effects on firm policies.

### The effects of flooding on CEO pay-performance sensitivity

We further study whether firms adjust their executive compensation policies in response to the flood threat. In particular, we focus on total CEO pay and CEO pay-performance sensitivity. Following the existing literature [39], we measure CEO pay-performance sensitivity by including an interaction term between firm performance and *Floodratio* in the following regression specification:

$${CEO\;pay}_{ict+1}=\alpha_{1}{Floodratio}_{ct}+\alpha_{2}{Floodratio*Performance}_{ict}+\alpha_{3}{Performance}_{ict}+\alpha_{4}{Size}_{ict}{+\alpha_{5}{Leverage}_{ict}+\alpha_{6}{Largest}_{ict}+\alpha_{7}Lnboard}_{ict}+\alpha_{8}{Indep}_{ict}+\alpha_{9}{SOE}_{ict}{{+\alpha }_{10}{OCF}_{ict}+\alpha_{11}Volatility}_{ict}+\alpha_{12}{Sales\;Growth}_{ict}{+\alpha_{13}{Market-to-book}_{ict}+\alpha_{14}CEO\;age}_{ict}+\alpha_{15}{CEO\;tenure}_{ict}+\alpha_{16}{GDP}_{ct}+\alpha_{17}{Income\;per\;capita}_{ct}+\alpha_{18}{GDP\;growth}_{ct}+{Firm}_{i}+{Year}_{t}+\varepsilon_{it}$$ (5)

In Eq (5), *CEO Pay* is the natural logarithm of total CEO compensation. *Performance* is the performance of the firm relative to its industry peers. We consider both ROA and ROS as firms’ financial performance measures. To capture firms’ relative performance, we subtract the respective industry medians from the raw financial performance measures. The results of estimating Eq (5) are reported in Table 9.

**Table 9:** Flooding, CEO pay and CEO pay-performance relationship. This table reports the regression results of the effects of flooding on total CEO pay and CEO pay-performance sensitivity. Dependent variable is the natural logarithm of the CEO total compensation. An interaction term between *Floodratio* and relative firm performance is included to measure the CEO pay-performance sensitivity. Relative firm performance proxies are measured by removing the industry median value in each year. Variable definitions are in Table A1 in the S1 Appendix. Robust standard errors are clustered at the city-year level and reported in parentheses. *, ** and *** indicate the significance levels at 10%, 5% and 1%, respectively.

Dependent	(1)	(2)	(3)	(4)	(5)	(6)
Variables	CEO pay	CEO pay	CEO pay	CEO pay	CEO pay	CEO pay
Floodratio	-0.759***	-0.821***	-0.757***			
	(0.203)	(0.178)	(0.184)			
Floodratio * ROA_adj		-17.905***				
		(4.413)				
Floodratio * ROS_adj			-3.256			
			(2.041)			
Flooddummy				-0.058***	-0.049***	-0.050***
				(0.007)	(0.007)	(0.007)
Flooddummy * ROA_adj					-1.239***	
					(0.142)	
Flooddummy * ROS_adj						-0.306***
						(0.051)
ROA_adj		0.907***			1.105***	
		(0.084)			(0.090)	
ROS_adj			0.248***			0.297***
			(0.035)			(0.038)
Size	0.234***	0.226***	0.229***	0.234***	0.224***	0.228***
	(0.007)	(0.008)	(0.008)	(0.007)	(0.008)	(0.008)
Leverage	-0.131***	-0.058*	-0.075**	-0.132***	-0.057*	-0.077**
	(0.030)	(0.031)	(0.031)	(0.030)	(0.031)	(0.031)
Largest	-0.180***	-0.195***	-0.188***	-0.184***	-0.199***	-0.190***
	(0.047)	(0.047)	(0.048)	(0.047)	(0.047)	(0.048)
Lnboard	0.159***	0.150***	0.153***	0.160***	0.149***	0.153***
	(0.033)	(0.034)	(0.034)	(0.033)	(0.034)	(0.034)
Indep	0.132	0.093	0.103	0.129	0.088	0.097
	(0.082)	(0.082)	(0.082)	(0.082)	(0.082)	(0.082)
SOE	-0.131***	-0.132***	-0.133***	-0.131***	-0.133***	-0.133***
	(0.024)	(0.024)	(0.024)	(0.024)	(0.024)	(0.024)
OCF	1.096***	0.945***	1.050***	1.099***	0.953***	1.056***
	(0.078)	(0.080)	(0.079)	(0.078)	(0.079)	(0.079)
Volatility	0.098	0.309	0.375	0.090	0.256	0.357
	(0.104)	(0.438)	(0.447)	(0.104)	(0.436)	(0.446)
Sales growth	-0.008	-0.000	-0.006	-0.009	0.000	-0.006
	(0.006)	(0.007)	(0.007)	(0.006)	(0.007)	(0.007)
Market-to-book	0.005**	0.012***	0.013***	0.005**	0.012***	0.013***
	(0.002)	(0.002)	(0.002)	(0.002)	(0.002)	(0.002)
CEO_age	0.182***	0.176***	0.178***	0.182***	0.179***	0.179***
	(0.030)	(0.030)	(0.030)	(0.030)	(0.030)	(0.030)
CEO_tenure	-0.003	-0.003	-0.003	-0.002	-0.003	-0.002
	(0.005)	(0.005)	(0.005)	(0.005)	(0.005)	(0.005)
Past stock returns	0.017***	0.009**	0.011**	0.017***	0.008*	0.011**
	(0.004)	(0.004)	(0.004)	(0.004)	(0.004)	(0.004)
GDP	-0.178	-0.153	-0.146	-0.245**	-0.227*	-0.213*
	(0.122)	(0.121)	(0.122)	(0.120)	(0.120)	(0.121)
Income per capita	0.281***	0.262***	0.257***	0.336***	0.325***	0.316***
	(0.099)	(0.097)	(0.097)	(0.096)	(0.094)	(0.095)
Past GDP growth	-0.104	-0.262***	-0.262***	-0.119	-0.304***	-0.294***
	(0.084)	(0.096)	(0.096)	(0.082)	(0.093)	(0.093)
Observations	27,383	26,392	26,392	27,383	26,392	26,392
Adjusted R-squared	0.825	0.828	0.827	0.825	0.829	0.828
Year fixed effect	Yes	Yes	Yes	Yes	Yes	Yes
Firm fixed effect	Yes	Yes	Yes	Yes	Yes	Yes

Column 1 reports the results without the interaction term, where we study the effect of flooding on total CEO pay. The coefficient estimate of *Floodratio* is significantly negative at the 1% level, indicating that flood-exposed firms tend to decrease total CEO pay. Columns 2 and 3 report the regression results of interacting *Floodratio* with industry-adjusted ROA and ROS, respectively. Our main interest is the coefficient of the interaction term. The coefficient estimate of the interaction term is negative across both specifications and is statistically significant at the 1% level in column 2 (the interaction term using industry-adjusted ROA), indicating that flood-exposed firms tend to decrease CEO pay-performance sensitivity.

Columns 4 to 6 repeat the previous three columns by replacing *Floodratio* with *Flooddummy*. *Flooddummy* is an indicator variable which equals 1 if the value of *Floodratio* is in the top quartile of the sample in each year and equals 0 otherwise. The results are quantitatively similar to the results reported in columns 1 to 3. In terms of economic magnitude, CEO pay is on average 6% lower for firms exposed to high flood risk (i.e., *Flooddummy* = 1), compared to other firms. Furthermore, for every 1 percentage-point increase in industry-adjusted ROA (ROS), the increase in CEO pay is on average around RMB 23,000 (RMB 5,700) lower for firms exposed to high flood risk, compared to other firms. The calculation logic for these economic magnitudes is as follows. The coefficient of Flooddummy*ROA_adj is -1.239 in column 5 of Table 9. Thus, for every 1 percentage-point increase in industry-adjusted ROA, the increase in total CEO pay is 1.24 percentage-point lower for firms exposed to high flood risk, which is around RMB 23,000 lower (i.e., 1.24%*1,856,775; the average total CEO compensation per annum in the sample is RMB 1,856,775). The same calculation logic applies to every 1 percentage-point increase in industry-adjusted ROS in column 6 of the table.

Taken together, these results indicate that firm managers bear personal risk of flooding, as reflected in lower CEO pay. Moreover, flood-exposed firms adopt lower CEO pay-performance sensitivity given that the inferior firm performance relative to their non-flood-affected industry peers is at least partly attributable to flooding that is outside of managerial control.

## The effects of flooding on local economic and employment growth

Because the impact of flooding on local firms’ performance may aggregate into an impact on the local economy, in this section we study the impact of flooding on both employment growth and GDP growth at the city level. We construct the following two dependent variables: *Employment growth* is the employment growth rate, defined as the change in annual employment number of the city from previous year, scaled by the employment number of the previous year; *GDP growth* is the GDP growth rate, defined as the change in annual GDP of the city from previous year, scaled by the GDP of the previous year.

In the regression analysis, we also include several time-varying, city-level control variables, including income per capita, GDP and past GDP growth, as well as city and year fixed effects. We cluster standard errors at the city level. Specifically, we estimate the following city-level regression specification:

$${Employment\;or\;GDP\;growth}_{ct+1}=\alpha_{1}{Floodratio}_{ct}+\alpha_{2}{GDP}_{ct}+\alpha_{3}{Income\;per\;capita}_{ct}+\alpha_{4}{Past\;GDP\;growth}_{ct}+{City}_{c}+{Year}_{t}+\varepsilon_{it}$$ (6)

Table 10 reports the regression results. For comparison, we report both OLS and 2SLS estimation results. Columns 1 and 2 report the OLS regression results using the two dependent variables, respectively. The results show that the coefficient estimates of *Floodratio* are negative and statistically significant at the 5% level in both regressions, suggesting that flooding significantly impacts local employment and economic growth. In terms of economic magnitudes, a one-standard-deviation increase in flood ratio is on average related to a 1.38-percentage-point decrease in employment growth and a 0.08-percentage-point decrease in GDP growth of the city.

**Table 10:** Flooding, GDP growth rate, and employee growth: City-level analysis. This table reports the city-level OLS regression results of the effects of flooding on local employee growth and GDP growth. The sample has 3,299 city-year observations, including those cities with at least one listed firm during the period of 2003 to 2019. Dependent variables are employment growth rate and GDP growth rate, both of which are measured at the city level. Other variables are also measured at the city level. Variable definitions are in Table A1 in the S1 Appendix. Robust standard errors are clustered at the city level and reported in parentheses. *, ** and *** indicate the significance levels at the 10%, 5% and 1%, respectively.

	(1)	(2)
Dependent Variables	Employment growth	GDP growth
Floodratio	-0.865**	-0.052**
	(0.359)	(0.027)
GDP	0.865*	0.120***
	(0.502)	(0.030)
Income per capita	-1.455***	-0.060**
	(0.502)	(0.028)
Past GDP growth	-0.654	-0.093*
	(0.798)	(0.053)
Constant	6.429	1.455***
	(5.392)	(0.129)
Observations	3,299	3,299
Adjusted R-squared	0.018	0.664
City fixed effects	Yes	Yes
Year fixed effects	Yes	Yes

To summarize, we document strong empirical evidence indicating that flooding exerts significant adverse impacts on local economic and employment growth. In particular, flood-affected cities experience significantly slower employment and GDP growth. These city-level empirical results complement the results from previous firm-level analyses. The findings suggest that flooding negatively affects firm performance, which in turn aggregates into an adverse impact on local economic and employment growth.

## Conclusion

In this paper, we study the impact of flooding on firm performance, firm policies and local economic growth using comprehensive flood data from China. China is a country with a vast geographic area, the largest population in the world, a relatively fast-growing economy, and large floods occurring almost every year. Thus, it provides us an ideal laboratory to empirically investigate and quantify the actual impact of flooding on firm performance, firm policies, and economic and employment growth.

Our analyses reveal a significant and negative impact of flooding on short-term future firm performance such as Tobin’s Q and return on assets. Our analyses also show that the significantly negative impact of flooding on firm performance is mainly driven by unexpected flooding and not by normal (well expected) flooding. Our empirical results from various identification strategies suggest that the negative impact of flooding on short-term future firm performance is most likely causal. Moreover, the impact of flooding on firm performance is more pronounced for firms with more intensive tangible asset investment, firms located in cities with low government quality, firms facing tight financial constraints, non-SOEs, and firms with low geographic diversification.

We further document that flood-exposed firms react to the threat by significantly altering their short-term investment, financial, cash, payout and executive compensation policies to mitigate the impact of flooding. Finally, we document a significantly negative and likely causal impact of flooding on local economic and employment growth.

Our findings contribute new empirical evidence to the currently scant literature on how flooding impacts firm performance, alters firm policies, and affects local economic growth. The findings suggest that flooding poses a threat to firm performance and economic growth. The magnitudes of the flooding impact on firm performance and economic growth are relatively modest, however. Our findings on the magnitudes of the flood impacts on firm performance and economic growth and the cross-sectional heterogeneity of the impacts could generate useful implications for insurance policy pricing, taxation and government subsidies, and infrastructural investments.

Our empirical findings further suggest that firms can mitigate the adverse impact of flooding on the firm’s performance through substituting intangible asset investment for tangible asset investment, increasing geographic diversification, and obtaining insurance policies. Nevertheless, because flooding is likely caused by climate changes and global warming, the long-run, sustainable solution should be to reduce fossil fuel usage and the associated CO_2_ emission to reach the goal of the Paris Agreement in curbing global warming below 1.5 degrees. The findings in this study may be of interest to investors, academics, and economic policy makers.

## Supporting information

S1 Appendix (DOCX)

S1 Data (DO)
