# Supplementary material for: The impact of flooding on firm performance and economic growth
Source: PLoS One. 2022 Jul 13;17(7):e0271309. doi: 10.1371/journal.pone.0271309 (PMC9278787; doi:10.1371/journal.pone.0271309)
Supplement: S1 Appendix — (DOCX) [file pone.0271309.s001.docx]

**Appendix**

**Table A1. Variable definition**

| Variable name | Definition and measurement |
| --- | --- |
| ***Flood area*** |  |
| Floodratio | The area of flooding / Total area of the city, measured at the city level |
| Floodratio_sudden | Equal to *Floodratio* if the value of *Floodratio* of a city in a year is at least two-standard-deviation larger than the average *Floodratio* of that city (where the firm’s headquarter is located) during our sample period and equal to 0 otherwise |
| Floodratio_normal | Equal to *Floodratio* if the value of *Floodratio* of a city in a year is *not* at least two-standard-deviation larger than the average *Floodratio* of that city during our sample period and equal to 0 otherwise |
| ***Firm performance*** |  |
| Tobin’s Q | (Market value of equity + book value of debt) / Total assets |
| Stock returns | Annual stock returns |
| ROA | Net income / Total assets |
| ROS | Net income / Total sales |
| ***Firm investment policies*** |  |
| R&D | R&D expenditure / Total assets |
| CAPEX | Capital expenditure / Total assets. Capital expenditure is the amount of investment in property, plant, and equipment. |
| MA | An indicator variable that equals 1 if the firm announces M&A transaction(s) in the year and equals 0 otherwise |
| MA_nonflood | An indicator variable that equals 1 if the firm announces M&A transaction(s) and the target firm is headquartered in a city which is not affected by the flooding in the year and equals 0 otherwise |
| MA_flood | An indicator variable that equals 1 if the firm announces M&A transaction(s) and the target firm is headquartered in a city which is affected by the flooding in the year and equals 0 otherwise |
| MA_exp | M&A expenses / Total assets |
| ***Firm financing policies*** |  |
| Book leverage | Total debt / Total assets |
| Market leverage | Total debt / (Book value of debt + market value of equity) |
| Debt maturity | Long-term debt / Total debt |
| Cash holding | Cash and cash equivalents / Total assets |
| ***Firm payout policies*** |  |
| Dividend | An indicator variable that equals 1 if firm pays cash dividends and equals 0 otherwise |
| DPS | Cash dividend per share |
| DPS/Book per share | Cash dividend per share / Book value of equity per share |
| Repurchase ratio | Repurchased value / Market value of equity |
| Repurchase | An indicator variable that equals 1 if firm buys back common shares outstanding and equals 0 otherwise |
| ***Managerial compensation*** |  |
| CEO pay | The natural logarithm of the CEO compensation, including the basic salary, bonus and other forms of cash compensation |
| ***Other variables*** |  |
| Market-to-book | Market value of equity / Book value of equity |
| Size | The natural logarithm of firm total assets |
| Largest | Percentage of ownership held by the largest shareholders |
| Ln(Board) | The natural logarithm of total number of board directors |
| Indep | Ratio of independent directors on the boards |
| Tangibility | Fixed assets / Total assets |
| Intangibility | Intangible assets / Total assets |
| SOE | An indicator variable that equals 1 if the ultimate owner of the firm is the government and equals 0 otherwise |
| OCF | Operating cash flows / Total assets |
| Sales growth | The growth rate of annual sales |
| CEO age | The age of the CEO |
| CEO tenure | The number of years as the firm’s CEO |
| Volatility | Standard deviation of daily stock returns of the previous year |
| Subsidiary | The number of the cities that subsidiaries of the listed firms are located |
| Employment growth | The change of employment number from previous year / employment number of previous year, measured at the city level |
| GDP growth | The change of GDP from previous year / GDP of previous year, measured at the city level |
| GDP | The natural logarithm of GDP (in millions RMB), measured at the city level |
| Income per capita | The natural logarithm of annual income per capita, measured at the city level |

**Table A2. Flooding and firm performance: Excluding Beijing, Shanghai, Shenzhen and Guangzhou**

This table reports the results of the effects of flooding on firm performance using various proxies for firm performance, including *Tobin’s Q* and *ROA*, which are measured in year *t+*1. We exclude firms headquartered in Beijing, Shanghai, Shenzhen and Guangzhou from the sample. The key independent variable is *Floodratio*, which is defined as the ratio of flood area in each city (where the firm’s headquarter is located) to the city’s total area and measured in year *t*. All of the control variables used in this table are also measured in year *t*. Variable definitions are in Table A1 in the Appendix. Robust standard errors are clustered at the city-year level and reported in parentheses. *, ** and *** indicate the significance levels at 10%, 5% and 1%, respectively.

| Dependent | (1) | (2) |
| --- | --- | --- |
| Variables | Tobin’s Q | ROA |
| Floodratio | -0.720* | -0.058** |
|  | (0.386) | (0.028) |
| Size | -0.860*** | -0.014*** |
|  | (0.052) | (0.001) |
| Leverage | 0.628*** | 0.013** |
|  | (0.095) | (0.006) |
| Largest | 0.151 | 0.055*** |
|  | (0.107) | (0.006) |
| Ln(board) | 0.188** | 0.001 |
|  | (0.073) | (0.005) |
| Indep | 0.180 | 0.008 |
|  | (0.206) | (0.013) |
| SOE | -0.166*** | -0.003 |
|  | (0.046) | (0.003) |
| OCF | 3.369*** | 0.287*** |
|  | (0.282) | (0.015) |
| Tangibility | -0.296*** | 0.014** |
|  | (0.088) | (0.006) |
| Cash holding | 0.621*** | 0.064*** |
|  | (0.108) | (0.005) |
| CEO age | 0.063 | -0.000 |
|  | (0.077) | (0.005) |
| CEO tenure | -0.066*** | 0.000 |
|  | (0.014) | (0.001) |
| Volatility | 2.268*** | 0.012* |
|  | (0.427) | (0.007) |
| Market-to-book | 0.099* | -0.000 |
|  | (0.060) | (0.001) |
| Past stock returns | 0.238*** | 0.003*** |
|  | (0.029) | (0.001) |
| GDP | 0.048 | -0.007 |
|  | (0.379) | (0.020) |
| Income per capita | 0.205 | 0.009 |
|  | (0.344) | (0.017) |
| Past GDP growth | -0.059 | 0.001 |
|  | (0.232) | (0.010) |
| Observations | 21,752 | 21,669 |
| Adjusted R-squared | 0.729 | 0.443 |
| Year fixed effects | Yes | Yes |
| Firm fixed effects | Yes | Yes |

**Table A3. Flooding and firm performance: Lewbel’s (2012) instrumental-variable regression approach**

This table reports the results of the effects of flooding on firm performance using instrumental-variable regression approach proposed by Lewbel (2012). Variable definitions are in Table A1 in the Appendix. Robust standard errors are clustered at the city-year level and reported in parentheses. *, ** and *** indicate the significance levels at 10%, 5% and 1%, respectively.

| Dependent | (1) | (2) |
| --- | --- | --- |
| Variables | Tobin’s Q | ROA |
| Floodratio | -1.001*** | -0.065* |
|  | (0.367) | (0.034) |
| Size | -0.513*** | 0.000 |
|  | (0.054) | (0.000) |
| Leverage | -0.138 | -0.015*** |
|  | (0.103) | (0.003) |
| Largest | 0.312*** | 0.020*** |
|  | (0.057) | (0.002) |
| Ln(board) | 0.216*** | 0.005** |
|  | (0.052) | (0.002) |
| Indep | 1.533*** | 0.001 |
|  | (0.196) | (0.008) |
| SOE | -0.153*** | 0.002* |
|  | (0.020) | (0.001) |
| OCF | 3.448*** | 0.465*** |
|  | (0.407) | (0.012) |
| Tangibility | -0.239*** | 0.018*** |
|  | (0.052) | (0.002) |
| Cash holding | 0.855*** | 0.051*** |
|  | (0.108) | (0.003) |
| CEO age | 0.092 | 0.003 |
|  | (0.067) | (0.003) |
| CEO tenure | -0.064*** | 0.002*** |
|  | (0.013) | (0.001) |
| Volatility | 3.114*** | -0.005 |
|  | (0.552) | (0.005) |
| Market-to-book | 0.194* | 0.000 |
|  | (0.102) | (0.001) |
| Past stock returns | 0.231*** | 0.002*** |
|  | (0.043) | (0.001) |
| GDP | -0.116*** | 0.002*** |
|  | (0.018) | (0.001) |
| Income per capita | 0.165*** | 0.002*** |
|  | (0.030) | (0.001) |
| Past GDP growth | 0.001 | -0.008 |
|  | (0.273) | (0.008) |
| Observations | 29,808 | 29,635 |
| Centered R-squared | 0.530 | 0.586 |
| Year fixed effects | Yes | Yes |
| Firm fixed effects | Yes | Yes |
| Weak instrument test: |  |  |
| Cragg-Donald Wald F statistic | 6643.43 | 7969.86 |
| Stock-Yogo crit. val. | 21.41 | 21.41 |

**Table A4. Flooding and firm performance: Alternative measures of performance**

This table reports the results of the effects of flooding on firm performance using alternative proxies for firm performance, including *Stock returns* and *ROS*, which are measured in year *t+*1. The key independent variable is *Floodratio*, which is defined as the ratio of flood area in each city (where the firm’s headquarter is located) to the city’s total area and measured in year *t*. All of the control variables used in this table are also measured in year *t*. Variable definitions are in Table A1 in the Appendix. Robust standard errors are clustered at the city-year level and reported in parentheses. *, ** and *** indicate the significance levels at 10%, 5% and 1%, respectively.

| Dependent | (1) | (2) |
| --- | --- | --- |
| Variables | Stock returns | ROS |
| Floodratio | -0.543*** | -0.162** |
|  | (0.201) | (0.063) |
| Size | -0.037*** | -0.017*** |
|  | (0.009) | (0.004) |
| Leverage | 0.249*** | -0.012 |
|  | (0.035) | (0.016) |
| Largest | 0.096** | 0.165*** |
|  | (0.043) | (0.020) |
| Ln(board) | -0.035 | 0.005 |
|  | (0.033) | (0.011) |
| Indep | -0.268*** | 0.032 |
|  | (0.086) | (0.032) |
| SOE | -0.018 | -0.002 |
|  | (0.017) | (0.009) |
| OCF | 0.966*** | 0.460*** |
|  | (0.072) | (0.037) |
| Tangibility | 0.085** | 0.005 |
|  | (0.039) | (0.016) |
| Cash holding | 0.018 | 0.181*** |
|  | (0.038) | (0.021) |
| CEO age | -0.045 | -0.010 |
|  | (0.035) | (0.017) |
| CEO tenure | 0.004 | 0.003 |
|  | (0.006) | (0.002) |
| Volatility | 0.953*** | 0.054** |
|  | (0.312) | (0.024) |
| Market-to-book | 0.009* | 0.005*** |
|  | (0.005) | (0.001) |
| Past stock returns | -0.072*** | 0.006*** |
|  | (0.008) | (0.002) |
| GDP | -0.091 | -0.076* |
|  | (0.119) | (0.044) |
| Income per capita | -0.048 | 0.052* |
|  | (0.100) | (0.029) |
| Past GDP growth | 0.571*** | 0.038 |
|  | (0.114) | (0.038) |
| Observations | 30,414 | 29,631 |
| Adjusted R-squared | 0.482 | 0.328 |
| Year fixed effects | Yes | Yes |
| Firm fixed effects | Yes | Yes |

**Table A5. Flooding and firm performance: Firms with versus without insurance compensation**

This table reports the results of the effects of flooding on firm performance using various proxies for firm performance, including *Tobin’s Q* and *ROA*, which are measured in year *t+*1. The key independent variable is *Floodratio*, which is defined as the ratio of flood area in each city (where the firm’s headquarter is located) to the city’s total area and measured in year *t*. All of the control variables used in this table are also measured in year *t*. In Panel A, we include only firms without insurance compensation in the regressions. In Panel B, we include only firms with insurance compensation in the regressions*.* Variable definitions are in Table A1 in the Appendix. Robust standard errors are clustered at the city-year level and reported in parentheses. *, ** and *** indicate the significance levels at 10%, 5% and 1%, respectively.

| Dependent | (1) |  | (2) |
| --- | --- | --- | --- |
| Variables | Tobin’s Q |  | ROA |
| **Panel A: Future performance of firms without insurance compensation** | | | |
| Floodratio | -0.765** |  | -0.056** |
|  | (0.352) |  | (0.024) |
| Observations | 28,988 |  | 28,807 |
| Adjusted R-squared | 0.729 |  | 0.445 |
| Control variables | Yes |  | Yes |
| Year fixed effects | Yes |  | Yes |
| Firm fixed effects | Yes |  | Yes |
| **Panel B: Future performance of firms with insurance compensation** | | | |
| Floodratio | 0.113 |  | 0.051 |
|  | (0.794) |  | (0.105) |
| Observations | 698 |  | 712 |
| Adjusted R-squared | 0.876 |  | 0.710 |
| Control variables | Yes |  | Yes |
| Year fixed effects | Yes |  | Yes |
| Firm fixed effects | Yes |  | Yes |

**Table A6. Flooding, contemporaneous firm performance, and long-term future firm performance**

This table reports the results of the effects of flooding on firms’ contemporaneous and long-term future performance using various proxies for firm performance, including *Tobin’s Q* and *ROA*. In Panel A, the firm performance variables are measured in year *t*. In Panel B, the firm performance variables are measured in year *t+*2. In Panel C, the firm performance variables are measured in year *t+*3*.* The key independent variable is *Floodratio*, which is defined as the ratio of flood area in each city (where the firm’s headquarter is located) to the city’s total area and measured in year *t*. All of the control variables used in this table are also measured in year *t*. Variable definitions are in Table A1 in the Appendix. Robust standard errors are clustered at the city-year level and reported in parentheses. *, ** and *** indicate the significance levels at 10%, 5% and 1%, respectively.

| Dependent | (1) |  | (2) |
| --- | --- | --- | --- |
| Variables | Tobin’s Q |  | ROA |
| Panel A: Contemporaneous performance in year *t* | | | |
| Floodratio | 0.113 |  | -0.002 |
|  | (0.450) |  | (0.005) |
| Observations | 26,446 |  | 27,444 |
| Adjusted R-squared | 0.737 |  | 0.954 |
| Control variables | Yes |  | Yes |
| Year fixed effects | Yes |  | Yes |
| Firm fixed effects | Yes |  | Yes |
| Panel B: Future performance in year *t+*2 | | | |
| Floodratio | -0.551 |  | -0.023 |
|  | (0.472) |  | (0.022) |
| Observations | 28,106 |  | 26,802 |
| Adjusted R-squared | 0.689 |  | 0.407 |
| Control variables | Yes |  | Yes |
| Year fixed effects | Yes |  | Yes |
| Firm fixed effects | Yes |  | Yes |
| Panel C: Future performance in year *t+*3 | | | |
| Floodratio | -0.260 |  | -0.028 |
|  | (0.540) |  | (0.025) |
| Observations | 25,006 |  | 23,763 |
| Adjusted R-squared | 0.677 |  | 0.416 |
| Control variables | Yes |  | Yes |
| Year fixed effects | Yes |  | Yes |
| Firm fixed effects | Yes |  | Yes |

**Table A7. Flooding and long-term future firm policies**

This table reports the regression results of the effects of flooding on long-term future firm policies. Dependent variables are R&D expenditures to total assets (*R&D*), capital expenditures to total assets (*CAPEX*), probability of conducting M&As (*MA*), M&A expenses to total assets (*MA_exp*), probability of conducting M&As in non-flooding city (*MA_nonflood*), probability of conducting M&As in flooding city (*MA_flood*), book leverage (*Book leverage*), market leverage (*Market leverage*), debt maturity (*Maturity*), cash holding levels (*Cash holding*), probability of paying cash dividends (*Dividend*), dividend per share (*DPS*), DPS to book value of equity per share (*DPS/Book per share*), repurchase payment to market value of equity (*Repurchase ratio*), and probability of repurchase (*Repurchase*). In In Panel A, the future firm-policy variables are measured in year *t+*2. In Panel B, the future firm-policy variables are measured in year *t+*3*.* The key independent variable is *Floodratio*, which is defined as the ratio of flood area in each city (where the firm’s headquarter is located) to the city’s total area and measured in year *t*. The control variables (also measured in year *t*) used in Tables 6, 7 and 8 are included but their coefficient estimates are omitted from reporting for brevity. Variable definitions are in Table A1 in the Appendix. Robust standard errors are clustered at the city-year level and reported in parentheses. *, ** and *** indicate the significance levels at 10%, 5% and 1%, respectively.

**Panel A: Firm policies in year *t+*2**

| Dependent | (1) | (2) | | | (3) | (4) | | | (5) | | | (6) |
| --- | --- | --- | --- | --- | --- | --- | --- | --- | --- | --- | --- | --- |
| Variables | R&D | CAPEX | | | MA | MA_exp | | | MA_nonflood | | | MA_flood |
| Floodratio | 0.001 | -0.002 | | | 0.022 | 0.002 | | | 0.027 | | | -0.006 |
|  | (0.004) | (0.025) | | | (0.167) | (0.013) | | | (0.160) | | | (0.111) |
| Observations | 16,408 | 28,188 | | | 21,928 | 21,928 | | | 21,928 | | | 21,928 |
| Adjusted R-squared | 0.838 | 0.308 | | | 0.240 | 0.240 | | | 0.199 | | | 0.197 |
| Control variables | Yes | Yes | | | Yes | Yes | | | Yes | | | Yes |
| Year fixed effects | Yes | Yes | | | Yes | Yes | | | Yes | | | Yes |
| Firm fixed effects | Yes | Yes | | | Yes | Yes | | | Yes | | | Yes |
| Dependent | (1) | | | (2) | | | (3) | | | (4) | | |
| Variables | Book leverage | | | Market leverage | | | Debt maturity | | | Cash holding | | |
| Floodratio | 0.022 | | | 0.043 | | | 0.053 | | | 0.005 | | |
|  | (0.057) | | | (0.047) | | | (0.047) | | | (0.035) | | |
| Observations | 23,986 | | | 27,125 | | | 27,158 | | | 23,980 | | |
| Adjusted R-squared | 0.768 | | | 0.811 | | | 0.622 | | | 0.591 | | |
| Control variables | Yes | | | Yes | | | Yes | | | Yes | | |
| Year fixed effects | Yes | | | Yes | | | Yes | | | Yes | | |
| Firm fixed effects | Yes | | | Yes | | | Yes | | | Yes | | |
| Dependent | (1) | | (2) | | (3) | | | (4) | | | (5) | |
| Variables | Dividend | | DPS | | DPS/Book per share | | | Repurchase ratio | | | Repurchase | |
| Floodratio | -0.079 | | -0.040 | | 0.004 | | | -0.000 | | | -0.435** | |
|  | (0.152) | | (0.072) | | (0.017) | | | (0.001) | | | (0.208) | |
| Observations | 27,146 | | 24,013 | | 25,502 | | | 27,146 | | | 27,146 | |
| Adjusted R-squared | 0.485 | | 0.563 | | 0.180 | | | 0.197 | | | 0.405 | |
| Control variables | Yes | | Yes | | Yes | | | Yes | | | Yes | |
| Year fixed effects | Yes | | Yes | | Yes | | | Yes | | | Yes | |
| Firm fixed effects | Yes | | Yes | | Yes | | | Yes | | | Yes | |

**Panel B: Firm policies in year *t+*3**

| Dependent | (1) | (2) | | | (3) | (4) | | | (5) | | | (6) |
| --- | --- | --- | --- | --- | --- | --- | --- | --- | --- | --- | --- | --- |
| Variables | R&D | CAPEX | | | MA | MA_exp | | | MA_nonflood | | | MA_flood |
| Floodratio | 0.006 | 0.072* | | | -0.066 | -0.005 | | | -0.058 | | | -0.008 |
|  | (0.004) | (0.043) | | | (0.192) | (0.015) | | | (0.149) | | | (0.120) |
| Observations | 14,855 | 25,055 | | | 19,291 | 19,291 | | | 19,291 | | | 19,291 |
| Adjusted R-squared | 0.843 | 0.301 | | | 0.248 | 0.248 | | | 0.205 | | | 0.204 |
| Control variables | Yes | Yes | | | Yes | Yes | | | Yes | | | Yes |
| Year fixed effects | Yes | Yes | | | Yes | Yes | | | Yes | | | Yes |
| Firm fixed effects | Yes | Yes | | | Yes | Yes | | | Yes | | | Yes |
| Dependent | (1) | | | (2) | | | (3) | | | (4) | | |
| Variables | Book leverage | | | Market leverage | | | Debt maturity | | | Cash holding | | |
| Floodratio | -0.010 | | | 0.007 | | | 0.020 | | | -0.064** | | |
|  | (0.067) | | | (0.049) | | | (0.041) | | | (0.028) | | |
| Observations | 21,102 | | | 23,986 | | | 24,023 | | | 21,100 | | |
| Adjusted R-squared | 0.771 | | | 0.805 | | | 0.632 | | | 0.604 | | |
| Control variables | Yes | | | Yes | | | Yes | | | Yes | | |
| Year fixed effects | Yes | | | Yes | | | Yes | | | Yes | | |
| Firm fixed effects | Yes | | | Yes | | | Yes | | | Yes | | |
| Dependent | (1) | | (2) | | (3) | | | (4) | | | (5) | |
| Variables | Dividend | | DPS | | DPS/Book per share | | | Repurchase ratio | | | Repurchase | |
| Floodratio | 0.094 | | -0.071 | | 0.014 | | | 0.001 | | | -0.244 | |
|  | (0.215) | | (0.063) | | (0.020) | | | (0.002) | | | (0.250) | |
| Observations | 24,013 | | 21,136 | | 22,579 | | | 24,013 | | | 24,013 | |
| Adjusted R-squared | 0.471 | | 0.578 | | 0.171 | | | 0.215 | | | 0.419 | |
| Control variables | Yes | | Yes | | Yes | | | Yes | | | Yes | |
| Year fixed effects | Yes | | Yes | | Yes | | | Yes | | | Yes | |
| Firm fixed effects | Yes | | Yes | | Yes | | | Yes | | | Yes | |
